# Adaptor protein Abelson interactor 1 in homeostasis and disease

**DOI:** 10.1186/s12964-024-01738-z

**Published:** 2024-10-01

**Authors:** Max Petersen, Pat Dubielecka

**Affiliations:** 1 Division of Hematology/Oncology, Department of Medicine, Warren Alpert Medical School of Brown University and Rhode Island Hospital, Providence, RI USA; 2 Center for the Biology of Aging, Brown University, Providence, RI USA; 3 Legoretta Cancer Center, Brown University, Providence, RI USA

**Keywords:** Abelson interactor 1, Adaptor protein, Protein complex, Signal transduction, Actin cytoskeleton, Endocytosis, Cancer, Pathogen infection, Development, Smooth muscle contraction, Inflammatory signaling, Centrosome regulation, Wnt signaling, Integrin signaling, Cell adhesion, Cell migration, Epithelial-mesenchymal transition, Proximity dependent labeling, Proximity proteomics, TurboID, Mass spectrometry

## Abstract

Dysregulation of Abelson interactor 1 (ABI1) is associated with various states of disease including developmental defects, pathogen infections, and cancer. ABI1 is an adaptor protein predominantly known to regulate actin cytoskeleton organization processes such as those involved in cell adhesion, migration, and shape determination. Linked to cytoskeleton via vasodilator-stimulated phosphoprotein (VASP), Wiskott-Aldrich syndrome protein family (WAVE), and neural-Wiskott-Aldrich syndrome protein (N-WASP)-associated protein complexes, ABI1 coordinates regulation of various cytoplasmic protein signaling complexes dysregulated in disease states. The roles of ABI1 beyond actin cytoskeleton regulation are much less understood. This comprehensive, protein-centric review describes molecular roles of ABI1 as an adaptor molecule in the context of its dysregulation and associated disease outcomes to better understand disease state-specific protein signaling and affected interconnected biological processes.

## Introduction

Actin cytoskeleton constitutes a tightly orchestrated scaffold needed for homeostasis of intracellular response. It is critical for adhesion, communication, membrane transport, migration, cell cycle, growth, and development. As a fundamental and dynamic cellular structure, dysregulated cytoskeleton organization is broadly associated with various pathologies where, for example, bacteria instruct cytoskeletal machinery to facilitate invasion, abnormal signal transduction between cytoskeleton and nucleus leads to oncogenesis and metastasis, and failures in cellular communications due to defective cytoskeletal action lead to immune, neurological, or developmental detriment. Cytoplasmic protein signaling cascades bridge cellular environment with cytoplasmic and nuclear response. Understanding molecular mechanisms and outcomes of cytoskeleton dysregulation is crucial to disease etiology and treatment.

**Fig. 1:**
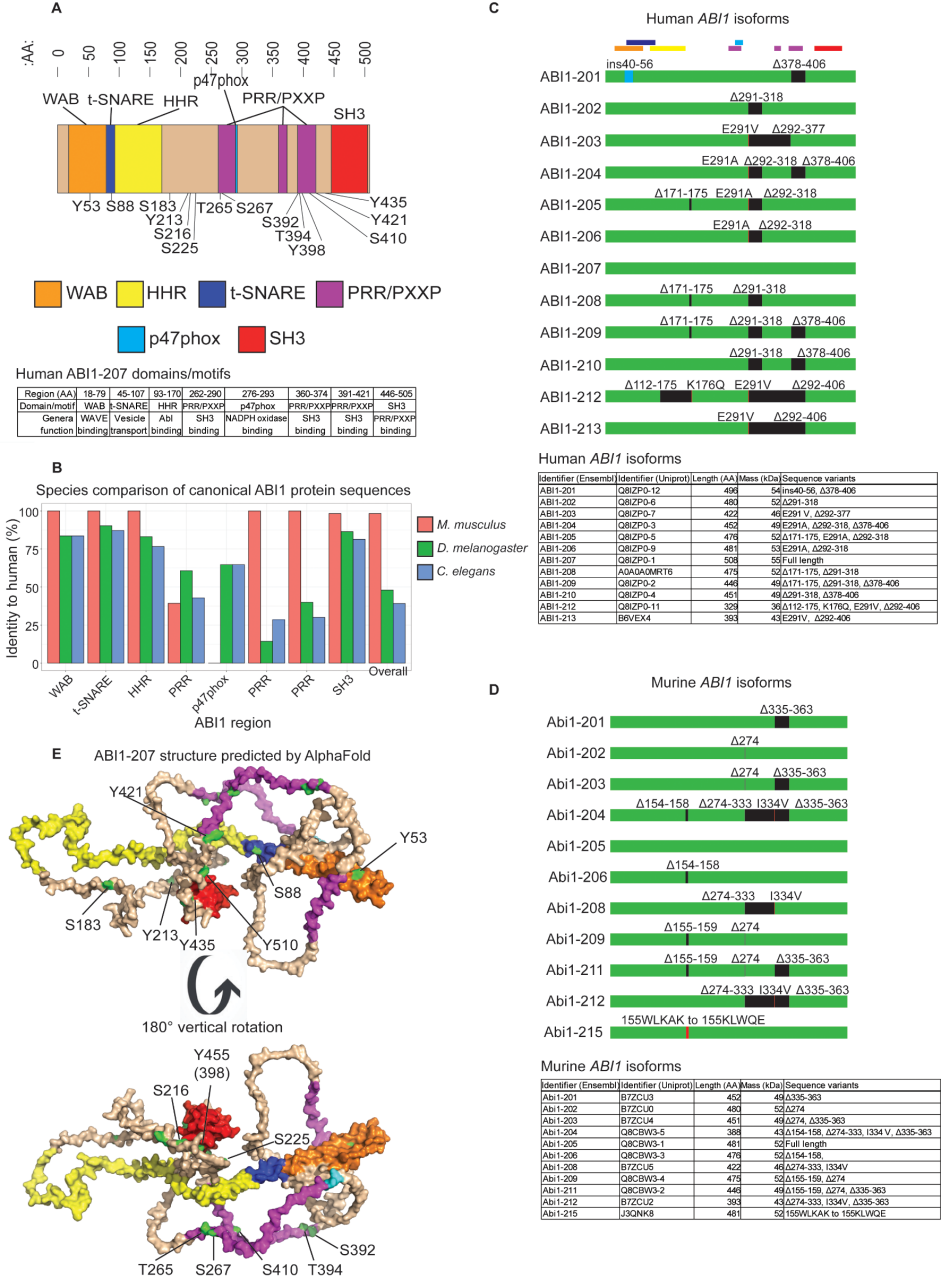
Summary of Abelson interactor 1 (ABI1) structure and isoforms. **(A)** Primary structure of ABI1, including domains and phosphosites. Orange: WAVE binding (WAB) domain. Dark blue: Target-Soluble *N*-ethylmaleimide-Sensitive Factor Attachment Proteins receptor (t-SNARE) domain. Yellow: Homeodomain homologous region (HHR). Purple: Proline rich region/Proline-X-X-Proline motif containing-domain (PRR/PXXP). Light blue: p47phox-binding region. Red: SRC homology 3 (SH3) domain. Regions in light brown are not assigned to a particular domain structure. An amino acid marker (:AA:) is included. Human ABI1-207 domains and motifs, including region and general function. **(B)** ABI1 canonical sequence comparison (semi-conserved substitutions accepted) by ABI1 region between human and mouse, fruit fly, and nematode **C)***Top*: Curated human ABI1 isoforms. ABI1-207 is considered full length ABI1. Multicolored bars above panel represent ABI1 domains and motifs, as indicated in Fig. 1A. *Bottom*: Curated human ABI1 isoforms, listing Ensembl identifier, Uniprot identifier, amino acid length, molecular weight, and sequence variants relative to full length ABI1 (ABI1-207). **D)***Top*: Curated murine ABI1 isoforms. ABI1-205 is considered full length ABI1. *Bottom*: Curated murine ABI1 isoforms, as in Fig. 3C, with sequence variants relative to full length ABI1 (ABI1-205). **C**,** D)** Regions in green represent protein-coding regions. Regions in black represent regions that are excluded from the isoform, relative to full length. Regions in red represent insertion/deletion events. Amino acid substitutions, deletions, and insertion/deletions are noted above each isoform. **E)** ABI1-207 structure predicted by AlphaFold. Phosphosites are labeled. Domains and motifs are indicated by coloration, as in Fig. 1A.

Abelson interactor 1 (ABI1) is a signal-facilitating adaptor protein that regulates cytoskeleton organization and response by coordinating various protein complex interactions. It was first discovered through a yeast two-hybrid screen using the Abelson kinase (ABL1) family-specific C-terminal region as bait [1]. Constitutive activation of ABL1 is a hallmark of certain cancers such as chronic myeloid leukemia (CML) and is a major focus of therapeutics development. ABI1 was also discovered independently as an epidermal growth factor receptor (EGFR) pathway substrate 8 (EPS8)- (E3B1) [2] and spectrin-binding protein- (SSH3BP1) [3], supporting its role in dynamic cytoskeleton reorganization linked to cytoplasmic signaling cascades. ABI1 is evolutionarily conserved, with orthologs present in most metazoans. ABI1 is abundant in cytoskeletal leading-edge protrusions and is also localized throughout the cytoplasm with distinct perinuclear localization [3, 4]. ABI1 was also reported to be detected at low levels in nuclear fractions [5]. ABI1 has several structural elements that facilitate its adaptive interactor function, including a C-terminal Src Homology 3 (SH3) domain [1], proline-rich regions (PRR) [1], PXXP motifs [1], a homeodomain homologous region (HHR) [1], WAVE binding domain [5], and a target-soluble N-ethylmaleimide-sensitive factor attachment protein receptor (T-SNARE) domain [5] (Fig. 1A). These functional domains are largely conserved across species (Fig. 1B). Multiple ABI1 isoforms are produced by alternative splicing in both human and murine tissues (Fig. 1**C, D**), and domain loss affected by these isoforms shows differential effects in disease signaling. Additionally, ABI1 has several phosphosites that are modified to affect signaling outcomes (Table 1). Structural prediction of ABI1 by AlphaFold supports its capabilities as an adaptor protein, indicated by positioning of protein interaction motifs and adjacent phosphosites (Fig. 1E). ABI1 crystal structures have yet to be established, in part due to its disordered regions and dynamic conformations that depend on specific binding partners, all in line with ABI1 adapter protein function. The critical roles of ABI1 in cytoskeleton organization and signal transduction, enabled by its protein interaction capabilities, are consistent with broad disease phenotypes associated with ABI1 dysregulation.

**Table 1:** Reported ABI1 phosphosites

Phosphosite	Source	Organism of discovery	Note
Y53	Proepper 2007	Rat	Required for nuclear entry with Myc/Max to promote neuronal synapse maturation.
Y213	Xiong 2008	Human	Phosphorylated by ABL to promote ABL binding and inhibition, dephosphorylated by PTEN (Qi 2020).
Y398	Sato 2011	Human	Promotes ABL binding, with Y213, predicted Y455 in ABI1-207.
Y421	Dubielecka 2011	Human	Phosphorylated form has high affinity for FYN SH2, weak binding to p85, no binding to ABL SH2.
Y435	Steinestel 2012	Human	Promotes matrix dissolution, fibronectin attachment, cell invasion.
Y506	Dubielecka 2011	Human	ABL SH2 binding site.
S88	Park 2012	Rat	Phosphorylated by CaMKIIa upon glutamate receptor activation to dissociate from Ca/CaM, essential for spine maturation.
S183	Mendoza 2011	Human	ERK phosphosite, plays role in WRC activation to promote ARP2/3 complex interaction and actin polymerization.
S216	Zhuang 2011	Mouse	Target of CDK/cyclin B kinase, phosphorylated at onset of mitosis, ablation reduced WAVE phoshorylation by BCR-ABL, mutants interfere with cell cycle progression, dephosphorylated by PTEN (Qi 2020).
S225	Mendoza 2011	Human	ERK phosphosite, plays role in WRC activation to promote ARP2/3 complex interaction and actin polymerization.
S267	Mendoza 2011	Human	ERK phosphosite, plays role in WRC activation to promote ARP2/3 complex interaction and actin polymerization.
S392	Mendoza 2011	Human	ERK phosphosite, plays role in WRC activation to promote ARP2/3 complex interaction and actin polymerization.
S410	Mendoza 2011	Human	ERK phosphosite, plays role in WRC activation to promote ARP2/3 complex interaction and actin polymerization.
T265	Mendoza 2011	Human	ERK phosphosite, plays role in WRC activation to promote ARP2/3 complex interaction and actin polymerization.
T394	Mendoza 2011	Human	ERK phosphosite, plays role in WRC activation to promote ARP2/3 complex interaction and actin polymerization.

**Fig. 2:**
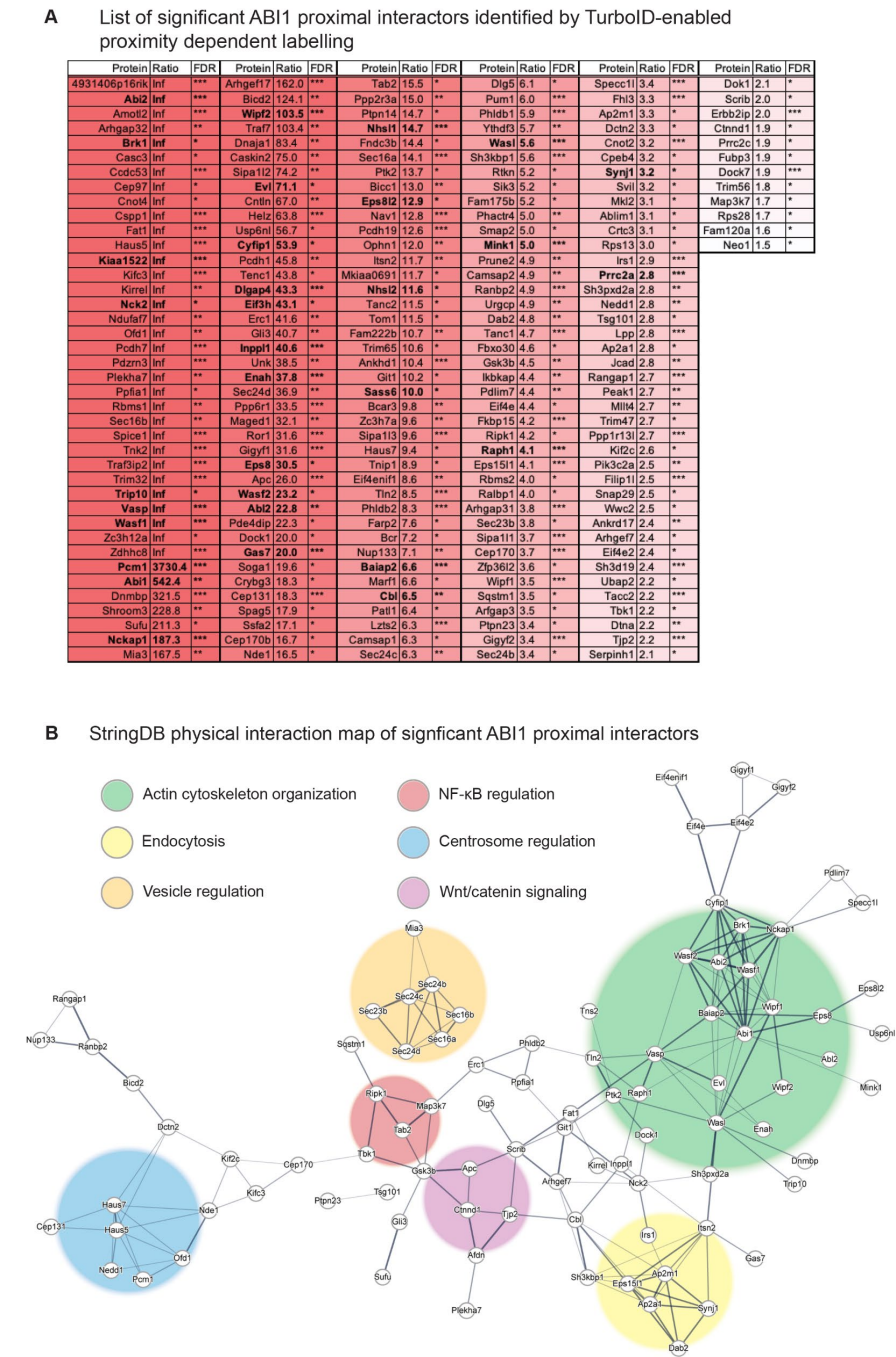
Significant Abelson interactor 1 (ABI1) proximal interactors measured in mouse embryonic fibroblast (MEF) cell proximity proteomics. **(A)** List of significant ABI1 proximal interactors measured in MEF cells, ordered by decreasing TurboID-ABI1 vs. TurboID protein abundance (PA) ratio. Rows are shaded by PA ratio, where darker red indicates higher PA ratio. * false discovery rate (FDR) ≤ 0.05, ** FDR ≤ 0.005, *** FDR ≤ 0.0005 for TurboID-ABI1 vs. TurboID PA. Bolded rows indicate known ABI1 interactors. **(B)** StringDB physical interaction map of significant ABI1 proximal interactors in MEF cells (TurboID-ABI1 vs. TurboID and untransduced controls PA and peptide spectrum match (PSM) fold-change ≥ 1.5 (FDR ≤ 0.05) and average PSM in TurboID-ABI1 ≥ 1). Nodes represent measured ABI1 proximal interactors and edges represent interaction confidence (interaction score (IS) 0.4-1.0), where thicker edges indicate higher IS. Shaded circles were manually added based on clusters of similar Gene Ontology Biological Process. Green circle represents actin cytoskeleton organization, yellow circle represents endocytosis, orange circle represents vesicle regulation, red circle represents NF-κB regulation, blue circle represents centrosome regulation, and purple circle represents Wnt/catenin signaling

In this review, we summarize known functions of ABI1 reported to date to better understand the broad pathological effect of ABI1 dysregulation in different contexts. We start with a description of mechanisms by which ABI1 regulates the actin cytoskeleton, followed by a review of the roles ABI1 plays in development, pathogen infection, smooth muscle contraction, and cancer. Throughout this review of ABI1-affected biological processes, we highlight ABI1-affiliated proteins identified by proximity proteomics in a recent report from our lab [6] to both bolster known mechanisms of regulation and support a more complete understanding of interaction networks underpinning dysregulated cellular processes of disease.

## ABI1 and actin cytoskeleton

Actin polymerization is initiated by different classes of actin nucleators working in concert to organize the cytoskeleton. Different mechanisms of actin nucleation function both separately and together to regulate formation of actin-dependent structures and enable cellular functions such as adherence, motility, and protein transport in response to both cell intrinsic and extrinsic stimuli. Actin nucleation is mediated by protein complex interactions that orchestrate dynamic cytoskeleton remodeling in response to activation of Rho family guanosine triphosphate hydrolases (GTPase) including Rat sarcoma virus (Ras)-related C3 botulinum toxin substrate 1 (RAC1), cell division control protein 42 homolog (CDC42), and RHOA [7]. Known types of actin nucleators include actin related protein 2/3 complex (ARP2/3), Ena/VASP, and formins. ABI1 is a critical regulator of actin nucleation through the coordination of protein complexes activating ARP2/3, Ena/VASP, and, under certain conditions, Diaphanous-related (Dia) formins. This section provides brief overviews of the ways ABI1 regulates actin cytoskeleton machineries.

### ARP2/3 complexes

ARP2/3 is activated by nucleation promoting factors such as the WAVE regulatory complex (WRC), a pentameric protein complex comprising ABI1, WAVE2, SRA1/CYFIP1, BRK1, and NCKAP1/NAP1 [8, 9]. GTP-bound RAC binds WRC to promote ARP2/3-mediated branched actin filament formation, forming sheet-like lamellipodia [8–10]. Loss of ABI1 induces loss of all WRC components by complex instability and degradation [10]. All WRC components were labeled as ABI1 proximal interactors (Fig. 2**AB)** [6], providing confidence that core ABI1 proximal interactors are capturable by proximity dependent labeling followed by mass spectrometry.

N-WASP also stimulates ARP2/3-mediated actin polymerization, activated by CDC42 and enhanced by phosphatidylinositol 4,5-bisphosphate (PIP2) [11]. ABI1 binding N-WASP induces its activity towards specific actin-regulated processes such as EGFR signaling and vesicle transport [12]. N-WASP was labeled as an ABI1 proximal interactor (Fig. 2**AB**) [6].

Ena/VASP family proteins directly elongate actin filaments in a progressive manner. They remain attached to the barbed end of the growing filament and are associated with formation of spiky filopodia actin structures [13]. Ena/VASP can also interact with WRC through ABI1 to enhance lamellipodia formation and migration [14]. VASP and associated proteins enabled homolog (ENAH) and Ena/VASP-like (EVL) were identified as ABI1 proximal interactors (Fig. 2**AB**) [6].

Finally, another key group of actin-regulating proteins, Dia formins, are activated by Rho, Rac, and CDC42 GTPases to also directly promote progressive actin polymerization [15]. Protein diaphanous homolog 1 (DIAPH1/mDia1) interacts with Ena/VASP and WRC, furthering mechanistic links regulating actin nucleation [16]. In the absence of WAVE, ABI1 interacts with mDia1, promoting β-catenin and E-cadherin-mediated cell-cell adhesion. This is independent of WRC-mediated ARP2/3 activation and requires ABI1 HHR region-mediated NAP1 binding [17]. Disheveled-associated activator of morphogenesis 1 (DAAM1), a Dia formin involved in non-canonical Wnt signaling, development, and neurogenesis [15], but not DIAPH1/mDia1, was identified as ABI1 proximal interactor (Fig. 2**AB**) [6], further supporting the role of ABI1 as a mediator of divergent signaling pathways.

## PI3K, EGFR, and EPS8/SOS1/ABI1 complex

ABI1 is a component of the EPS8/son of sevenless homolog 1 (SOS1)/ABI1 complex, which coordinates RAC GTPase activation upstream of WRC activation in response to extracellular stimuli [8, 18–20]. ABI1 plays a multi-regulatory role in this context, enabling signaling crosstalk to affect processes including phosphoinositide 3-kinase (PI3K), EGFR, WRC-mediated cytoskeletal remodeling, and protein kinase B (AKT)-mediated cell survival and proliferation pathways dysregulated in cancer [21–26]. PI3K inhibitory subunit p85 interacts with EPS8/SOS1/phosphorylated ABI1 to induce RAC activation, enhanced by phosphatidylinositol (3,4,5)-triphosphate (PIP3) [27]. ABL-directed ABI1 Y213 phosphorylation is also required for binding to p85 and is associated with repression of macropinocytosis [28]. ABL inhibition disrupts formation of β-catenin-associated cell junctions [29], and PI3K inhibition promotes cell junction formation associated with increased levels of β-catenin, E-cadherin, and glycogen synthase kinase 3 beta (GSK3β) activation [30], together indicating divergent signaling adapted by ABI1. Furthermore, ABI1 Y213 phosphorylation negatively regulates WRC through Casitas B-lineage lymphoma (CBL) E3 ubiquitin ligase-mediated ABI1 proteolysis [26]. CBL is a known interactor of ABI1 that also functions to negatively regulate EGFR signaling by promoting receptor endocytosis [31] dependent on interaction between ABI1 and N-WASP [12]. CBL homolog CBL-B also downregulates EGFR [32]. CBL is also involved in EPS8 degradation mediated through adaptor protein intersectin 1 (ITSN1), perturbing EPS8/SOS1 function and RAC activation [33]. Together, this begins to illustrate intricate cross regulations among actin organization machineries moderating cellular outcomes, mediated through ABI1 and intracellular signaling molecules. EPS8, PIK3C2A, CBL, ITSN1, IQGAP1 (adaptor protein affecting EGFR), but not SOS1, were labeled as ABI1 proximal interactors (Fig. 2**AB**) [6]. Notably, pleckstrin homology (PH) domains, which are involved in actin cytoskeleton reorganization events at the plasma membrane and have high affinity for phosphoinositides, were significantly overrepresented in the groups of ABI1 proximal interactors [6], reinforcing the role of ABI1 in PI3K regulation (Fig. 2**AB**).

### Endocytosis and vesicle trafficking

Endocytosis is a process dependent on actin cytoskeleton reorganization, and ABI1 plays roles in actin-associated protein and vesicle trafficking through multiple modes of regulation including CDC42-N-WASP interactions [12]. Actin dynamics also affect integrity and function of the Golgi apparatus, a series of compartments and microtubule organizing center involved in modification and vesicular trafficking of nascent proteins. Interestingly, ABI1 and WAVE, but not WASP, regulate Golgi stack separation required for mitotic entry [34], linking ABI1 activity in this context to broader cellular processes such as cell cycle. Several proteins involved in vesicle trafficking from the endoplasmic reticulum to the Golgi apparatus were identified as ABI1 proximal interactors including protein transport protein 16 A (SEC16A), SEC16B, SEC23B, and SEC24B, among others (Fig. 2**AB**) [6]. Adenosine diphosphate (ADP)-ribosylation factor 1 (ARF1) GTPase regulates endosomes via CDC42-N-WASP-mediated ARP2/3 activation, and promotes WRC activation via SRA1 interaction [35]. ARF1 is also involved in recruiting AP-1 and clathrin to trans-Golgi network membranes, which in turn promotes recruitment of ABI1, NAP1, and SRA1 to initiate RAC-N-WASP-ARP2/3-mediated tubule formation that affects Golgi dynamics. This is further regulated by GTPase effectors Rho guanine nucleotide exchange factor 7 (ARHGEF7/β-PIX) and ARF GTPase-activating proteins GIT1/2 [36]. β-PIX, GIT1, GIT2, ARFGAP3 (a GTPase-activating protein (GAP) of ARF1) and stromal membrane-associated protein 2 (SMAP2) were identified as ABI1 proximal interactors (Fig. 2**AB**) [6]. It must also be noted that RAC1 was not detected as a proximal interactor of ABI1, despite evidence supporting direct interaction between RAC1 and ABI1 [27, 37]. This might be explained by the transient nature of GTPases in regulating protein complex activation, and how this might be reflected in different cell types by frequency of cytoskeletal regulation events.

### Integrin signaling

ABI1 also plays a role in regulating cell migration through interaction with proteins that interface with the cellular environment. Integrin ⍺4 phosphorylation and subsequent RAC1 activation is restricted to the leading edge of migrating cells, promoting localized lamellipodia formation and directional motility [7, 38]. Integrin adhesion promotes recruitment of RAC GEF dedicator of cytokinesis protein 1 (DOCK1), breast cancer resistance protein 1 (BCAR1/p130Cas), and CRK, leading to cell spreading and divergent downstream signaling [39]. ABL inhibits this complex formation and resulting cell spreading by phosphorylating CRK [40]. ABI1-deficient K562 cells, which express CML fusion protein breakpoint cluster region (BCR)-ABL, show an integrin signaling-mediated quiescence phenotype characterized by increased integrin ⍺4 expression, resistance to BCR-ABL inhibitor imatinib, decreased proliferation, and increased adhesion associated with elevated p130Cas-CRK activation [41]. Cortactin and p130Cas are Src family kinase (SFK) substrates that contain SH2 domains, and these proteins are tyrosine phosphorylated in response to integrin engagement, linked to SFKs focal adhesion kinase (FAK/PTK2) and CRK activation to promote actin polymerization [42, 43]. Integrins including integrin ⍺3, integrin ⍺5, and integrin β1 were not detected whereas FAK, DOCK1, p130Cas, and cortactin were among identified proximal interactors [6]. In a separate mode of regulation independent from DOCK1-p130Cas-CRK, paxillin (PXN) inhibits lamellipodia formation by binding to unphosphorylated integrin ⍺4 at non-protrusive cell peripheries. This recruits ARF GAP GIT1, attenuating ARF activation and inhibiting RAC1 activation. This inhibition can be alleviated through activity of ARF guanine nucleotide exchange factors (GEF) ARNO and PIX, further supporting ARF-mediated RAC regulation to localize protrusion formation to the leading edge [38]. In addition to GIT1 and β-PIX being detected as ABI1 proximal interactors (Fig. 2**AB**), ARNO was also detected [6]. Given the known role of ABI1 in promoting lamellipodia formation, it is not surprising that ABI1 also seems to regulate mechanisms of lamellipodia inhibition in mouse embryonic fibroblast (MEF) cells. As in vesicle trafficking, this mechanism is likely attributed to an adaptor role of ABI1 in mediating GEF and GAP, as well as SFK coordination with different regulatory complexes. Together, this posits ABI1 instruction of integrin ⍺4 signaling outcomes as a coupling mechanism between migration and adhesion.

### ABLs and ABIs

Abelson kinase 1 (ABL1) is involved in both actin cytoskeleton regulation and inflammatory signaling through cytoplasmic protein interactions [44, 45], and these activities are regulated by ABI1. The SH3 domain of ABI1 is indispensable for binding of ABI1 to ABL1 [1], primarily through ABL1 residues P634 and P781 [46]. Loss of SH3-mediated negative regulation of ABL1 is associated with unchecked cell division and leukemia [47]. Pathogenic roles of ABL1 are further discussed in the *ABI1 in cancer* section of this review. Interestingly, ABL2 but not ABL1 was highly labeled by ABI1 proximity labeling in MEF cells [6] (Fig. 2**AB**). This might be attributed to broader cellular localization of ABL1 compared to ABL2, which is primarily associated with cytoskeleton [48]. ABL2 promotes formation of adhesion-dependent lamellipodial protrusions, binding cortactin SH3 domain via ABL2 PXXP motifs [49] and colocalizing with PXN and integrin β3 [48]. This is dependent on the C-terminal region of ABL2 [557-C]. ABL family proteins share similar N-terminal regions, including SH3, SH2, and kinase domains, while C-terminal regions are distinct [48]. Supporting this, protein sequence alignment between ABL1 (P00519) and ABL2 (P42684) N-terminal region [1-556] shows 89% identity, whereas alignment between ABL1 and ABL2 C-terminal region [557-C] shows 27% identity. This suggests a regulatory mode of ABI1, distinct from its regulation of ABL1, that acts through ABL2 C-terminus to affect protein signaling downstream of actin cytoskeleton events.

Finally, ABI2 and ABI3 are adaptor proteins with similar domain architecture and high sequence similarity to ABI1 [1, 50, 51]. ABI1 (Q8IZP0) and ABI2 (Q9NYB9) protein sequences are more similar to each other than to ABI3 (Q9P2A4), sharing 74% identity but only 47% and 45% identity with ABI3, respectively. ABI3 is shorter than ABI1 and ABI2, with full length isoforms comprising 366 amino acids compared to 508 and 513 amino acids, respectively. This is reflected in function, as ABI2 shows a similar role to ABI1 in ABL regulation [50] and likely acts in a compensatory capacity, while ABI3 appears to compete with ABI1 for WRC binding to negatively regulate actin nucleation [52]. Binding of ABI3 to WRC is negatively regulated by PI3K/AKT phosphorylation [53], linking integrin signaling and adhesion to this mechanism of actin polymerization regulation. Furthermore, while ABI3 retains HHR, PRR, PXXP, and SH3, it does not promote ABL phosphorylation as do ABI1 and ABI2 [54]. Loss of ABI1 is linked to increased expression of ABI2 [10], consistent with a compensatory function. ABI2 but not ABI3 was labeled as an ABI1 proximal interactor [6], further supporting distinct function of ABI3 from ABI1 and ABI2 (Fig. 2**AB**).

Dynamic interactions coordinated through ABI1 enable tight control of cellular response. ABI1 is a well conserved cornerstone of actin cytoskeleton regulation and further maintains cellular homeostasis by transducing signals inward. ABI1 is an adaptor protein with broad effects on signaling and cellular outcome, and its proximal interactions may indicate targetable mechanisms of disease. The following sections detail biological impacts of ABI1 signaling, which are largely mediated through its role in actin cytoskeleton organization but also through regulation of some components of cytoplasmic signaling.

### ABI1 in smooth muscle contraction

ABI1 regulates smooth muscle contraction through interaction with N-WASP to affect actin polymerization. ABI1 interacts with N-WASP in human airway smooth muscle (HASM) cells treated with contraction-stimulating acetylcholine. CDC42-mediated activation of N-WASP is dependent on ABI1. Acetylcholine stimulation promotes formation of an N-WASP-activating protein complex comprising ABI1, ABL1, and p130Cas. Formation of this complex, activation of ABL1 by Y412 phosphorylation, and subsequent actin polymerization and contractile force are attenuated by *ABI1* shRNA knockdown. Knockdown of *p130Cas* or *Abl1* also attenuate formation of the ABI1-ABL1-p130Cas complex [55], which were earlier discussed as ABI1 proximal interactors. Furthermore, p130Cas-related protein breast cancer anti-estrogen resistance protein 3 (BCAR3), which is also an interactor of CDC42, was labeled as ABI1 proximal interactor (Fig. 2**AB**) [6]. ABI1 is essential to initiate smooth muscle contraction by this complex and this is supported by proximity proteomics data.

Smooth muscle contraction is also regulated by association of actin-binding proteins profilin and cortactin, dependent on ABL1 [56]. In response to acetylcholine stimulation, ABI1 is acetylated at K416 by p300 acetyltransferase, promoting ABI1 association with and activation of N-WASP and subsequent smooth muscle contraction [57]. In smooth muscle cells, ABI1 recruits profilin to leading edges, promoting cell migration in a manner dependent on binding between profilin and the proline rich region of ABI1. *ABI1* knockdown in HASM cells reduces recruitment of profilin, ABL1, and activated N-WASP to the cell leading edge. Cortactin recruitment to the leading edge is unaffected by *ABI1* knockdown [58], consistent with previous studies showing that cortactin-profilin mediated cell migration is independent of ABI1 [56]. VASP recruitment to the leading edge is also unaffected by *ABI1* knockdown. ABI1 and profilin recruitment to the cell leading edge are reduced upon knockdown of ABL1 and β-integrin [58]. Together, these studies indicate parallel signaling pathways that enable contraction and migration in the absence of ABI1. Smooth muscle contraction mediated by ABI1 involves β-integrin, CDC42, p130Cas, profilin, ABL1, and N-WASP, while ABI1-independent mechanisms involve cortactin and VASP, further linking actin cytoskeleton and integrin regulation.

### ABI1 in development

#### Embryonic development

ABI1 has a critical role in embryonic development by regulating directional cell migration and adhesion. ABI1 knockout is associated with murine embryonic lethality due to malformations in the developing heart and brain, linked to reduced levels of WRC and decreased cell migration rate and distance [10]. Murine ABI1 knockout shares phenotypes with integrin ⍺4 knockout, characterized by midgestational lethality due to placental and cardiovascular abnormalities, associated with impaired cell spreading and ABI1 N-terminal binding to integrin ⍺4 [59].

ABI1 is also involved in *Caenorhabditis elegans* (*C. elegans*) morphogenesis and cell migration. In this context, ABI1 coordinates transducer of CDC42-dependent actin assembly (TOCA) family protein interaction with WAVE2 at cell junctions to affect actin dynamics and membrane trafficking [60]. The murine homolog of TOCA1, formin-binding protein 1-like (FNBP1L), as well as TOCA2 homolog family protein formin-binding protein 1 (FNBP1), are involved in cell migration, adhesion, and endocytosis. FNBP1 and FNBP1L have SH3 domains and Rho-interacting regions [61–63] that might bind ABI1 PRR and PXXP motifs to coordinate ARP2/3 activation through N-WASP and WRC. FNBP1 interactor formin-like protein 1 (FMNL1) can bind Rac and RhoA GTPases [64, 65], as well as SH3 domains [66]. FNBP1 was recently found to affect tumor survival, invasion, and metastasis through FAK/PI3K/AKT/mammalian target of rapamycin (mTOR) signaling [67], drawing connection between altered levels of cellular activity seen in both development and cancer. Additionally, ABL1 plays a critical role in embryonic morphogenesis of *Drosophila melanogaster* [68], and together this may be linked to phosphorylation of ABI1 promoting PI3K activation [22]. ABI1 affects the orchestration of tissue development by regulating several protein complexes controlling cell motility through actin cytoskeleton reorganization mechanisms. Furthermore, ABI1-mediated developmental processes are also dysregulated in cancer, suggesting developmental origins of systemic programs activated in malignancy.

ABI1 also plays an important role in *C. elegans* gonad development through interaction with WRC. The migratory path of gonadal distal tip cells (DTC) determines *C. elegans* gonad morphology. This depends on cytoskeleton reorganization to enable engulfment of apoptotic cells by phagocytic cells, driving migration of DTCs. Multiple pathways act in parallel to regulate WRC-mediated engulfment. These include cell death abnormality protein 1 (CED-1)/scavenger receptor class F member 1 (SCARF1) pathway activation of dynamin [69], and activation of CED-10/RAC GTPase [70]. Mechanisms inhibiting WRC-mediated engulfment include ABL1 inhibition of ABI1 [71] and a parallel pathway dependent on the tyrosine kinase binding domain of suppressor of lineage defect 1 (SLI-1), the *C. elegans* homolog of mammalian CBL [72]. As discussed earlier, CBL forms a complex with ABI1 in response to EGFR activation in human cell lines [31]. Binding of CBL to EGFR is required for EGFR endocytosis and inhibition during mitosis [73], consistent with the role of ABI1 in mediating N-WASP-dependent EGFR endocytosis [12] and further linking ABI1 activity to cell cycle regulation. SLI-1/CBL-mediated engulfment inhibition, dysfunction of which can lead to an abnormal number of gonad arms due to dysregulated DTC migration, is independent of CED-1/SCARF1, CED-10/RAC1, and ABL1 pathways [72]. This suggests a WRC-independent mechanism of DTC migration in the developing *C. elegans* gonad, involving SLI-1. An abnormal number of gonad arms is also observed in worms with mutations in CED-5/DOCK1 [72], which was earlier described as an ABI1 proximal interactor in the context of integrin-linked adhesion through DOCK1-RAC-p130Cas-CRK-ABL-mediated regulation of WRC activation (Fig. 2**AB**) [6]. This malformation is rescued by SLI-1/CBL loss-of-function mutant but not by ABI1 loss-of-function mutant [72].

### Neuronal development

Polarized outgrowth of axons is critical to directional cell migration and nervous system development. This process is regulated by actin cytoskeleton response to external guidance cues. In early neuronal development, ABI1 and ABL1 localize to growth cone particles and synaptosomes of projection neurons [74, 75]. ABI1 antagonizes filopodial outgrowth by forming a complex with N-WASP and small conductance calcium-activated potassium channel 3 (SK3), which is highly expressed in neural stem cells in early stages of neural stem cell differentiation, as well as in spines and postsynaptic densities of developing primary hippocampal neurons [76]. In later developmental stages, ABI1, EPS8, and SOS1 are enriched in dendritic spines and post synaptic densities where ABI1 interacts with scaffold protein SH3 and multiple ankyrin repeat domains 3 (SHANK3) [74]. At post synaptic densities, SHANK3 interacts with β-PIX, GIT1/2, and Scribble proteins to regulate actin-mediated recruitment of synaptic vesicles [77–80]. ABI1 downregulation by RNA interference in rat hippocampal neurons shows excessive dendritic branching, immature spine and synapse morphology, and reduced number of synapses [74]. While ABI1 proximity proteomics did not identify SHANK3 as a proximal ABI1 interactor, β-PIX, GIT1/2, and Scribble proteins were labeled (Fig. 2**AB**) [6].

Together, these reports support a role of ABI1 in regulating neuronal development, suggesting that ABI1 dysregulation might be associated with neurodevelopmental disease through postsynaptic vesicle recruitment. SHANK3 mutations are associated with autism spectrum disorder, and SHANK3 S782A mutation increases SHANK3 enrichment at excitatory synapses in hippocampal neurons [81]. SHANK3 is phosphorylated by known ABI1 interactor calcium/calmodulin-dependent protein kinase II-alpha (CaMKIIa) [81]. ABI1 interacts with CaMKII via the ABI1 t-SNARE domain, inhibiting both CaMKII activity and ABI1-dependent RAC1 activation. This inhibition is relieved by glutamate receptor activation followed by ABI1 S88 phosphorylation by CaMKII [82]. Glutamate receptor activation by N-methyl-D-aspartate (NMDA) induces ABI1 translocation from post synaptic densities to nuclei, dependent on ABL1 activity [74]. This indicates an ABI1-mediated signaling mechanism that transduces extracellular signals into the cell to affect neuronal development. Hyperactive formation of dendritic protrusions is also observed in mouse hippocampal neurons lacking dysbindin, a schizophrenia susceptibility gene and known ABI1 interactor [83, 84]. ABI1 in cells lacking dysbindin binds more CaMKII, inhibiting CaMKII phosphorylation in synaptosomal fractions. Decreased CaMKII activity in cells lacking dysbindin is associated with hyperactive dendritic protrusion activity and altered spine morphology, which is rescued by ABI1 overexpression through actin regulation mechanisms independent of CaMKII [84]. Together, these data suggest feedback mechanisms involving parallel roles of ABI1 that regulate post-synaptic density protrusions and may contribute to neurological disorder.

The MIG-10/Ras-related protein 1 (Rap1)-GTP-interacting adaptor molecule (RIAM)/Lamellipodin (LPD) (MRL) family of adaptor proteins also plays an important role in transmitting external guidance cues to the actin cytoskeleton machinery during neuronal development to promote axonal outgrowth and neuronal migration [85–87]. *C. elegans* MIG-10/LPD is an ABI1 interactor dependent on the SH3 domain of ABI1 to bind active RAC1 and regulate WRC [88]. Loss or perturbation of either MIG-10/LPD or ABI1 causes axon guidance defects, and interaction between MIG-10 and ABI1 is mediated through scaffold protein and known ABI1 interactor uncoordinated-53 (UNC-53) [89, 90]. Notably, UNC-53 ortholog neuron navigator 2 (NAV2) mutation is associated with human and mouse neurodevelopmental defects, linked to perturbation of migration and cytoskeletal regulation [91], and is also associated with Alzheimer’s disease [92]. This functional interaction regulating lamellipodia formation is conserved in frog, fly [88], and mammalian cells [89]. MIG-10 murine ortholog LPD/RAPH1 was identified as an ABI1 proximal interactor (Fig. 2**AB**) [6]. Together, these studies describe the spectrum of developmental outcomes affected by adaptor proteins such as ABI1, underlined by its fundamental role in regulating actin cytoskeleton and tissue-specific binding partner stoichiometry.

### ABI1 in infection

Pathogens transmit their own proteins across host cell membranes to support invasion, replication, and immune evasion. Transmitted proteins can interact with ABI1 or ABI1-interacting proteins to influence ABI1-regulated processes including cytoskeleton organization and cytoplasmic signal transduction. Various pathogens have evolved to hijack ABI1-targeted mechanisms that support pathogenicity, consistent with the essential role ABI1 plays in regulating fundamental cellular processes. The following subsections detail the major mechanisms by which ABI1 exploitation mediates infection.

### WRC-mediated invasion

*Listeria monocytogenes* internalization is induced by interactions between bacterial effector proteins and host proteins driving WAVE-, N-WASP-, and Ena/VASP-mediated cytoskeleton reorganization. Secreted *Listeria monocytogenes* protein InlB induces ruffle formation and phagocytic entry, and ABI1 localizes to sites of bacterial entry. ABI1 knockdown and consequential WRC disruption in HeLa and Vero cells inhibits *Listeria monocytogenes* invasion, indicating a role of ABI1 in facilitating invasion [66].

ABI1 also localizes to sites of *Salmonella* infection. RNA interference against *ABI1* disrupts WRC and impairs *Salmonella* internalization by HeLa cells [93]. Additionally, enhanced ABI1 phosphorylation by ABL1 is observed in response to *Salmonella* infection. Knockdown of *ABL1* and *ABL2* by RNA interference, or ABL1 pharmacological inhibition by imatinib, impairs *Salmonella* invasion of MEF or HeLa cells [94]. The focal adhesion proteins FAK and p130Cas, which regulate adhesion signaling with ABI1 [55, 95–97], function as protein complex scaffolds that signal onto PI3K [98, 99]. FAK and p130Cas are required for actin cytoskeleton reorganization to facilitate *Salmonella* invasion of MDCK and MEF cells [100], and this likely involves ABL1 activation of ABI1 to promote PI3K activation. FAK and p130Cas were earlier described as ABI1 proximal interactors (Fig. 2**AB**) [6].

Chlamydiae also exploit actin cytoskeleton rearrangement to facilitate invasion by activating RAC1 GTPase and WRC activity. Knockdown of either WAVE2 or ABI1 abrogates chlamydia-induced actin recruitment driven by WRC [101]. Upon host cell attachment, invading chlamydia bacteria secrete translocated actin recruiting protein (TARP) across the host plasma membrane into the cytoplasm, where it is rapidly phosphorylated. Phosphorylated TARP binds RAC GEFs SOS1 and VAV2 in a phosphotyrosine-dependent manner. Binding of phosphorylated TARP to SOS1 versus VAV2, a possible functional redundancy, depends on specific tyrosine phosphorylation and, in the case of VAV2, PI3K activation and availability of PIP3 substrate. Interaction of TARP with SOS1/ABI1/EPS8 complex is abolished upon *ABI1* knockdown by siRNA. *ABI1* knockdown shows greater reduction of invasion efficiency than knockdown of *EPS8*, *SOS1*, or *VAV2* in HeLa cells. ABI1 binds TARP oligopeptides containing either phosphotyrosine utilized by SOS1 or VAV2 GEFs [102].

Finally, human cytomegalovirus (HCMV) exploits host cytoskeleton reorganization machinery to disrupt cell-cell contacts necessary for immune attack by NK and T cells. HCMV protein pUL135 is a driver of HCMV pathogenic effect [103]. pUL135 interacts with ABI1 and ABI2 to recruit WRC to the plasma membrane. WRC recruitment to the plasma membrane induces actin cytoskeleton remodeling that reduces immune synapse formation. Additionally, interaction between pUL135 and Talin, a host protein that binds actin and is localized to focal adhesions, disrupts cell contacts with the extracellular matrix (ECM) [103]. pUL135 also perturbs EGFR signaling to facilitate HCMV infection, dependent on interactions between pUL135 and binding motifs on ABI1 and SH3-domain kinase-binding protein 1 (SH3KBP1) [104]. Talin and SH3KBP1 also promote integrin activation – Talin through association with PXN [105], and SH3KBP1 by suppressing protein phosphatase 2A (PP2A) activity [106]. ABI1 and SH3KBP1 are pUL135 interactors [104]. SH3KBP1 and Talin were labeled as ABI1 proximal interactors (Fig. 2**AB)** [6]. These studies demonstrate that direct binding of bacterial proteins to ABI1 can facilitate invasion through perturbation of normal actin cytoskeleton dynamics, further linking protrusion and adhesion mechanisms including EGFR and integrin signaling.

### Kinase involvement

Host ABL1 signaling is exploited by *Anaplasma phagocytophilum*, the tick-borne pathogenic agent causing granulocytic anaplasmosis. Phosphorylation of *Anaplasma* ankyrin-rich protein AnkA is necessary for invasion. AnkA interacts with ABI1, and inhibition of *ABL1* by siRNA knockdown or by imatinib impairs infection. Together, this suggests that *Anaplasma phagocytophilum* invasion depends on AnkA binding to ABI1 and subsequent ABL1 pathway activation [107].

Hepatitis C virus (HCV) affects EGFR signaling to promote viral replication, mediated through interaction of viral nonstructural protein 5 A (NS5A) with host ABI1. NS5A binds ABI1 in the cytoplasm, and ABI1 overexpression promotes HCV replication. Consistently, HCV replication is impaired in ABI1 knockout cells. Expression of NS5A inhibits EGFR-mediated activation of extracellular signal-regulated kinase (ERK) and early growth response factor 1 (EGR1), which promotes HCV replication [108, 109]. Neither EGR1 nor ERK proteins were enriched in proximity proteomics, suggesting an upstream role of ABI1 linked to EGFR complex regulation. Together, these data indicate a mechanism by which HCV exploits host signaling to promote its replication through ABI1.

Altogether, these studies of ABI1 and ABI1 signalome exploitation by pathogens highlight the fundamental and conserved role of ABI1 in mammalian cytoskeletal organization. ABL-family protein ordered regions are well conserved through evolution [110, 111], so it is fitting that bacteria produce proteins able to directly bind and exploit the function of ABI1 to promote invasion. ABI1 is a central player in cytoskeleton dynamics, and pathogens evolved independent mechanisms to take advantage of these processes to facilitate their invasion and propagation. Additionally, these studies demonstrate the range of different mechanisms by which ABI1 regulates actin cytoskeleton and cytoplasmic response.

### ABI1 in cancer

**Fig. 3:**
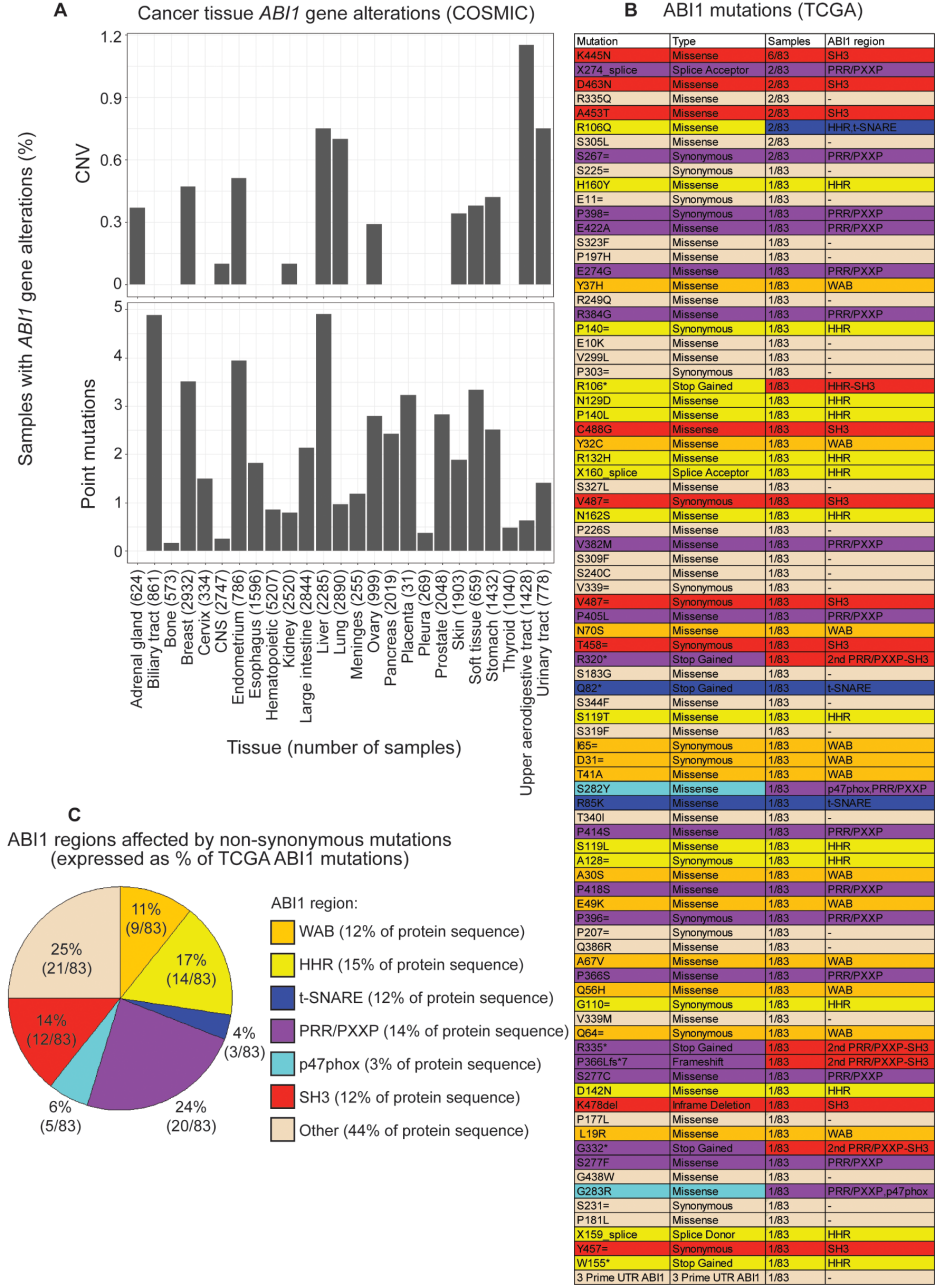
Abelson interactor 1 (ABI1) gene alterations reported in human cancers. **(A)** ABI1 gene alterations curated from Catalogue of Somatic Mutations in Cancer (COSMIC) database. Cancer tissue is indicated, followed by number of samples in parentheses. *Top*: Percentage of samples with ABI1 copy number variations (CNV), by cancer tissue. *Bottom*: Percentage of samples with ABI1 point mutations, by cancer tissue. **(B)** List of ABI1 mutations curated from The Cancer Genome Atlas (TCGA), including mutation, type of mutation, number of samples, and region of ABI1. Coloration is by region of ABI1, where orange represents WAVE binding domain, yellow represents HHR domain, blue represents t-SNARE domain, purple represents PRR/PXXP regions, light blue represents p47phox domain, red represents SH3 domain, and light brown represents regions not assigned to a structured domain. **(C)** Pie chart illustrating ABI1 regions affected by non-synonymous mutations listed in Fig. 3B. Coloration is as indicated in the legend. Percentages within the pie chart indicate frequency of mutation out of 83 observed ABI1 mutations in TCGA, and percentages within the legend indicate the amino acid content assigned to the listed structure relative to full length ABI1

ABI1 plays a complex role in cancer signaling, and both downregulation and upregulation are observed across cancer types. While individual studies may correlate ABI1 expression level with malignancy, these findings are convoluted by aggregate reporting, partially due to differences in method or material tested. ABI1 expression level in cancer patients is also associated with differences in 5-year survival, and in some cancers associated with cancer stage. Furthermore, while ABI1 point mutations, copy number variations, and even oncogenic fusion proteins are observed in cancer patients, they are rare (Fig. 3A). 83 ABI1 mutations were curated from The Cancer Genome Atlas (TCGA), though the majority of these are observed in only a single patient (Fig. 3B). Of recurrent ABI1 mutations, most are found in SH3 domain (Fig. 3B). While most observed non-synonymous mutations (25%) are not in ordered regions of ABI1, these regions make up 44% of the protein sequence and so are less mutated than expected. T-SNARE mutations are also less frequently observed than expected, while PRR/PXXP and p47phox regions are more frequently mutated than expected (Fig. 3C). Together, these data provide further evidence that ABI1 dysregulation is associated with malignancy and indicate regions of ABI1 critical for function and signaling homeostasis.

**Fig. 4:**
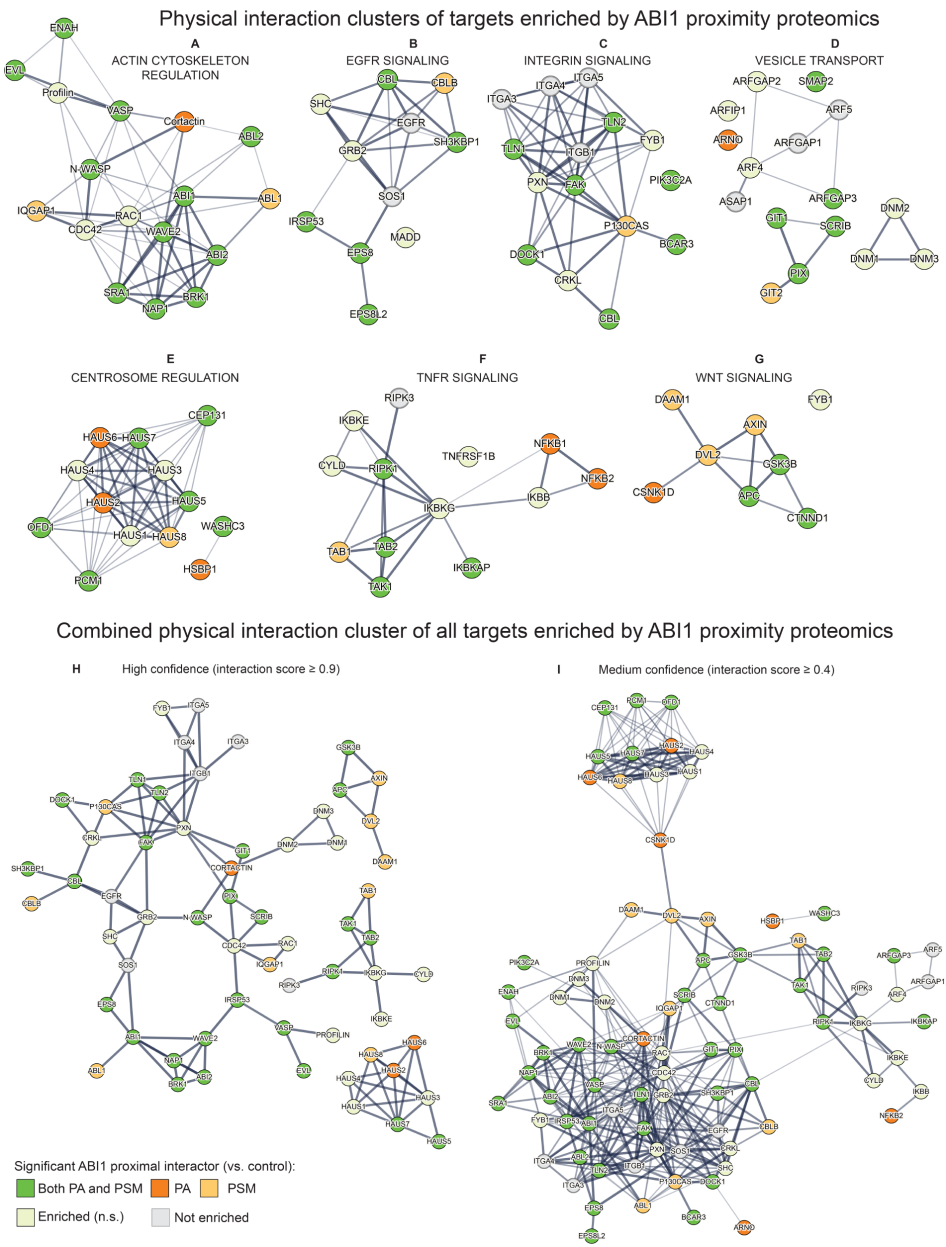
StringDB physical interaction maps of Abelson interactor 1 (ABI1) proximal interaction clusters based on mouse embryonic fibroblast (MEF) cell proximity proteomics, Gene Ontology Biological Process analysis, and literature review. Green circles represent proteins significantly enriched as ABI1 proximal interactors for both protein abundance (PA) and peptide spectrum match (PSM). Dark orange circles represent proteins significantly enriched as ABI1 proximal interactors by PA but not by PSM. Light orange circles represent proteins significantly enriched as ABI1 proximal interactors by PSM but not PA. Light yellow circles represent proteins enriched versus controls but not significant. Grey circles represent proteins that were not enriched versus control but are pertinent to the interaction cluster. Edges represent StringDB interaction score (IS 0.4–0.9), where thicker edges represent higher IS. **(A)** ABI1 proximally interacting proteins associated with actin cytoskeleton regulation. **(B)** ABI1 proximally interacting proteins associated with epidermal growth factor receptor (EGFR) signaling. **(C)** ABI1 proximally interacting proteins associated with integrin signaling. **(D)** ABI1 proximally interacting proteins associated with vesicle transport. **(E)** ABI1 proximally interacting proteins associated with centrosome regulation. **(F)** ABI1 proximally interacting proteins associated with tumor necrosis factor ⍺ receptor (TNFR) signaling. **(G)** ABI1 proximally interacting proteins associated with Wnt signaling. **(H)** Aggregate StringDB physical interaction map of proteins shown in Fig. 4A-G, with interaction score ≥ 0.9. **(I)** Aggregate StringDB physical interaction map of proteins shown in Fig. 4A-G, with interaction score ≥ 0.4

Accumulated literature indicates that ABI1 may act as a direct or indirect driver of malignancy affecting several different modes of complex protein regulation. In the following sections, the mechanistic roles ABI1 plays in cancer signaling are discussed to highlight both reported divergent and convergent activities of ABI1. Links among protein signal transduction processes, mediated by adaptor proteins such as ABI1, provide a pharmacologically relevant level of detail necessary to deconvolute cancer cell development and persistence.

### ABI1, ABL1 and BCR-ABL1

BCR-ABL1 is an oncogenic fusion protein resulting from t(9;22) chromosomal translocation in human hematopoietic stem cells, forming the Philadelphia chromosome. Fusion of *BCR* and *ABL1* genes produces BCR-ABL1, a constitutively active protein kinase that is the oncodriver of CML. The Abelson murine leukemia virus (v-Abl) causes a CML-like disease in mice similar to that caused by murine BCR-ABL1 infection [112]. Imatinib is a tyrosine kinase inhibitor that targets BCR-ABL1 and eliminates cells that are dependent on its activity. Imatinib is an effective treatment for CML, and a large majority of patients with CML who continuously take imatinib have a normal life expectancy. However, upon imatinib discontinuation, there is about 40% incidence of cancer relapse [113]. ABI1 is both a potent activator [114] and suppressor of ABL1 kinase activity, dependent on the mode of regulatory signaling [1, 115, 116]. In hematopoietic cells expressing oncogenic BCR-ABL or v-SRC, ABI1 is downregulated by the ubiquitin-proteasome pathway [117], which might be either a compensatory response to BCR-ABL1 overactivity (consistent with an activating role of ABI1), or a driver of disease progression (consistent with a suppressing role of ABI1). The roles of ABI1 in BCR-ABL1 signaling seem to be multi-faceted, indicating a far more complex signal transduction network of BCR-ABL1-driven disease than is currently understood. Therefore, ABI1 must be considered within a context of functional regulation beyond stoichiometry. Interestingly, BCR was labeled as a proximal ABI1 interactor (Fig. 2**AB**) [6].

ABI1 has a general capability to promote substrate phosphorylation by ABL1, resulting in activation or inhibition of various downstream cellular processes, including those regulating cell adhesion. ABI1 Y213 and Y398 phosphorylation promote ABL1 binding and activation, associated with enhanced adhesion of leukemic K562 or Ba/F3 cells expressing BCR-ABL1 [46]. It was also shown that VASP and Ena are phosphorylated by ABL1 or BCR-ABL1 in an ABI1 dependent manner, and that ABI1-mediated maintenance of the phosphorylation/dephosphorylation cycle of VASP Y39 by BCR-ABL1 is necessary for K562 adhesion to fibronectin [118]. BCR-ABL1 also forms focal adhesion complexes together with RAP1 GEF C3G, Crk-like protein (CRKL), p130Cas, CBL and ABI1 through SH3 interactions, dependent on ABI1 and p130Cas. Interestingly, knockdowns of C3G, CBL, or ABI1 decrease fibronectin adhesion, while p130Cas knockdown enhances fibronectin adhesion [96]. In CML, CBL is heavily phosphorylated by BCR-ABL and binds tyrosine phosphorylated CRK SH2 domain [119], which is associated with formation of a RAC1-activating complex [120, 121]. Notably, ABI1 deficiency in BCR-ABL positive cells is associated with quiescence, characterized by increased integrin-mediated adhesion and decreased migration [41]. Neither RAP1 nor C3G, but VASP, CRKL, p130Cas, and CBL were labeled as proximal interactors of ABI1 (Fig. 2**AB**) [6]. The role ABI1 plays in regulating cell adhesion through BCR-ABL1 interaction may contribute to malignant behavior of cancer cells, including propensity to metastasize and resist treatment. Understanding mechanistic details of how this is regulated might identify new and more generalizable therapeutic strategies.

As an adaptor protein, ABI1 can coordinate protein complex interactions between ABL1 and its substrates through several domain interactions, contextualized by domain availability and phosphorylation status. The SH3 domain of ABI1 interacts with the carboxy terminal region of v-ABL and suppresses its transforming ability by blocking ABL1 activation [1, 115]. Deletion or inactivation of the ABI1 SH3 domain abrogates interaction with the proline-rich region of ABL1 and inhibits ABL1-mediated phosphorylation of ABI1 [46]. In another example, the ABI1 SH3 domain binds the C-terminal polyproline region of phosphoinositide 3-kinase adaptor protein 1 (PIK3AP1), promoting ABL1 phosphorylation of five well-conserved C-terminal tyrosine residues [122]. ABL1 Y89 phosphorylation by SFKs such as HCK blocks ABI1 binding and its negative regulation of ABL1 [123]. Src kinase-associated phosphoprotein 2 (SKAP2) is an adaptor protein for SFKs that interacts with WAVE2 and cortactin, antagonizing their adhesion-induced translocation to the membrane and inhibiting glioblastoma cell migration [124]. SKAP2 has an N-terminal PRR, a PH domain, and a C-terminal SH3 domain, suggesting direct binding between ABI1 and SKAP2. SKAP2-ABI1 interaction may function as a mechanistic hub commuting ARP2/3, PI3K-AKT, SFK and ABL1 activity. ABL1 SH2 and SH3 domains bind concurrently with ABI1 pY213 and PXXP motif, respectively – a high affinity binding mechanism [3, 116, 125]. Imatinib treatment decreases ABI1 pY213, decreases co-immunoprecipitation of ABL1 by ABI1, and decreases ABL1-activating Y412 phosphorylation [116, 125]. ABL1 contains an allosteric myristoyl binding site that inhibits kinase activity [126], and therapeutics have been developed that target this site to stabilize an inactive conformation [127, 128]. Binding of ABI1 to a nonmyristoylated form of ABL1 is associated with decreased ABL1 Y412 phosphorylation in Cos7 cells. Additionally, BCR-ABL1 oligomerization, which is required for trans-activation and transforming capability, is affected by oligomeric ABI1 binding [129].

ABI1 depletion studies further illustrate the importance of ABI1 in regulating outcomes of BCR-ABL1 signaling. CRISPR/Cas9-mediated knockout of *ABI1* in BCR-ABL1-transformed hematopoietic cells impairs leukemogenesis in recipient mice, characterized by reduced interleukin-3 (IL3)-independent growth, reduced stromal cell-derived factor 1 alpha (SDF-1a)-mediated chemotaxis, reduced invadopodia formation, and reduced signaling of PI3K/AKT and ERK pathways. This *ABI1* knockout also impedes leukemogenesis of imatinib-resistant hematopoietic cells expressing BCR-ABL1 [130]. Furthermore, imatinib-resistant K562 cells show decreased ABI1 expression associated with increased levels of integrin ⍺4. This is also observed in relapsing BCR-ABL1^+^ CD34^+^ hematopoietic cells, associated with an anchorage dependent phenotype and increased activation of AKT and ERK [41]. ABI1 knockdown in BCR-ABL1^+^ Ba/F3 cells injected into nonobese diabetic/severe combined immunodeficiency (NOD/SCID) mice impairs leukemogenesis linked to decreased competitive expansion of cells expressing BCR-ABL1. While this ABI1 knockdown does not appear to affect total BCR-ABL1-induced tyrosine phosphorylation or IL-3 independent growth, BCR-ABL1^+^ Ba/F3 cells treated with *ABI1* shRNA show decreased formation of invadopodia, decreased migration, decreased LYN SFK activation, and decreased adhesion associated with membrane type 1 matrix metalloproteinase (MT1*-*MMP) clustering [131]. MT1-MMP1 promotes ECM degradation facilitating invadopodia formation, and its recruitment to F-actin-rich cytoskeleton structures is a key step in cell migration. Polarized recruitment of MT1-MMP to F-actin-rich structures is disrupted by ABI1 knockdown, linked to loss of ABI1 interaction with BCR-ABL1 p185 SH3 and C-terminal regions [132]. ABI1 also localizes to invadopodia in MDA-MB-231 breast cancer cell lines, and ABI1 knockdown impairs invadopodia formation, inhibits tumor cell proliferation and migration, decreases SRC activation, and downregulates matrix metalloproteinase 9 (MMP9) expression [133]. The apparent dichotomy of ABI1 expression on ABL1 activation and leukemic outcomes is likely due to the adaptive role of ABI1 in coordinating many protein interactions in signaling hubs maintaining cellular homeostasis (Fig. 2**AB**).

### ABI1 in centrosome regulation

Interaction between ABI1 and ABL1 also affects centrosome organization during mitosis, a commonly dysregulated process in cancer. ABI1 and ABL1 co-expression in Drosophila S2 cells (Schneider 2 cells derived from a primary culture of late-stage *Drosophila melanogaster* embryos, likely from a macrophage-like lineage) suppresses cell growth, linked to inactivation of cell division cycle protein 2 homolog (CDC2) by ABI1 complex coordination and ABL1-mediated phosphorylation of CDC2 Y15 [134]. CDC2 interactor budding uninhibited by benzimidazoles 1 (BUB1), a mitotic checkpoint protein that localizes to centromeres of mitotic chromosomes [135], is downregulated in response to BCR-ABL1 expression [136]. Actin cytoskeleton dynamics were recently shown to affect centrosome regulation [137]. In this process, ABI1 might play a cell cycle-regulated role in centrosome organization through pericentriolar material 1 (PCM1)-dependent recruitment of heat shock binding factor protein 1 (HSBP1) and coiled-coil domain containing 53 (WASHC3/CCDC53). These recruit the pentameric Wiskott-Aldrich syndrome protein and SCAR homolog (WASH) complex, comprising WASHC1-5, to the centrosome to initiate actin polymerization [138]. This process is regulated by cell-cycle dependent phosphorylation of PCM1 by polo-like kinase 4 (PLK4), which drives recruitment of PCM1 to centrosomes to initiate mitotic spindle assembly [139]. Notably, centrosome proteins including PCM1, centrosomal protein 131 (CEP131), oral-facial-digital syndrome 1 (OFD1), WASHC3, and human Augmin like complex subunit (HAUS) proteins, which are involved in centrosome and spindle integrity [140], were identified as ABI1 proximal interactors (Fig. 2**AB**) [6]. Altogether, these studies suggest an actin polymerization-linked regulatory role of ABI1 in mitotic centrosome organization through cell cycle-dependent WASH complex coordination, which may contribute to oncogenesis when perturbed.

### ABI1 in NF-κB regulation

Nuclear factor kappa-light-chain-enhancer of activated B cells (NF-κB) activation promotes survival, malignant gene transcription, and plays an essential role in BCR-ABL1 positive leukemias [45]. Additionally, hematopoietic stem/progenitor cells from patients with primary myelofibrosis (PMF) show decreased ABI1 and increased NF-κB activation. This is further recapitulated in mice with bone marrow specific ABI1 depletion [141]. There are several signaling cascades that promote NF-κB activation, including mitogen activated protein kinase kinase kinase 7 (MAP3K7)/transforming growth factor beta (TGFβ)-activated kinase (TAK1). While NF-κB activation through TAK1 is independently parallel to NF-κB activation resulting from BCR-ABL1 activity [142], a role of ABI1 in regulating TAK1-mediated NF-κB activation was recently described from ABI1 proximity proteomics data [6]. Promotion of cell survival through NF-κB activation can be initiated by tumor necrosis factor alpha (TNFα) receptor signaling. Upon binding of TNFα to its receptor, a cytoplasmic receptor complex forms that recruits TAK1-binding protein 1 (TAB1), TAB2, and TAK1, promoting autophosphorylation of TAK1, subsequent phosphorylation of inhibitor of κB kinase (IKK), and downstream activation of NF-κB. Opposing this survival signal, TNFα stimulation can also lead to cell death by promoting autophosphorylation of receptor interacting serine/threonine-protein kinase 1 (RIPK1) at S166, which leads to caspase cleavage and apoptosis. RIPK1 S321 phosphorylation, mediated by TAK1 and MAPK proteins, represses RIPK1 S166 phosphorylation and functions as an anti-apoptotic signal [143, 144]. ABI1 overexpressing cells show decreased RIPK1 S321 phosphorylation, and ABI1 deficient cells show general upregulation of RIPK1 and NF-κB pathway components. Upon TNFα stimulation, ABI1 deficient cells are protected from apoptosis, and this protection is negated in response to pharmacological inhibition of TAK1 by its inhibitor takinib. Furthermore, ABI1 deficient cells show decreased levels of RIPK1 S166 phosphorylation [6]. Together, these data link ABI1 to regulation of the balance of cell death and survival mediated by TNFα receptor signaling, specifically by repressing anti-apoptotic RIPK1 S321 phosphorylation by TAK1. RIPK1, TAK1, TAB, and other proteins involved in TNFα receptor signal transduction were labeled as ABI1 proximal interactors (Fig. 2**AB**).

### MLL-ABI1 fusion

The mixed-lineage leukemia 1 (MLL1) gene (lysine [K]-specific methyl transferase 2A (*KMT2A*)) located on chromosome 11q23 is disrupted in acute myeloid leukemias (AML), with more than 80 different *KMT2A* fusion partner genes described to date. Although rare, fusions between MLL and ABI1 are observed in infant AML patients with t(10;11)(p11.2;q23) translocation [145], suggesting a recurrent genetic event leading to *in utero* leukemogenesis by MLL-ABI1 fusion. Intriguingly, N-terminally truncated MLL1 alone is not sufficient to transform cells [146]. This argues for a crucial contribution on the part of the fusion partner, in this case t-SNARE domain-truncated ABI1, to leukemogenesis. Pediatric AML patients with MLL-ABI1 fusion show better prognosis and response to chemotherapy compared to AML patients presenting with other MLL fusions [145, 147–149]. Further assessment of MLL-ABI1 fusion activity can provide insight to broader signaling patterns that initiate leukemogenesis. MLL also fuses with extra eleven nineteen (EEN), a SH3 domain containing protein involved in endocytosis. EEN shows dynamic localization during hematopoietic cell cycle, colocalizing with the bipolar spindle during metaphase and anaphase [150], indicating a potential coregulatory role with other centrosome-regulating proteins identified as ABI1 proximal interactors. Fusion of EEN with MLL promotes leukemogenesis through HoxA7 promoter activation [151]. ABI1 and EEN both interact with dynamin and synaptojanin through SH3 domains, and ABI1 competes with EEN for synaptojanin binding [104]. Syanptojanin, involved in endocytosis and synaptic vesicle recycling, is expressed in bone marrow and immature hematopoietic progenitor cell lines [152]. Epidermal growth factor receptor substrate 15 (EPS15), which acts downstream of active EGFR, is also a MLL fusion partner and interactor of synaptojanin. Another AML-associated MLL fusion partner, mixed-lineage leukemia; translocated to, 1 (MLLT1/ENL), is also an ABI1 binding protein measured by yeast two-hybrid analysis and co-immunoprecipitation [153]. MLLT4/Afadin, which is involved in cell-cell adhesions downstream of Ras activation, is also an MLL fusion partner [154, 155]. Afadin is tyrosine phosphorylated by ABL1 to stabilize epithelial cell adherens junctions in Drosophila embryos [156]. Afadin interacts with cadherin-associated protein catenin delta-1 (CTNND1) to regulate adherens junctions [157]. High CTNND1 is associated with poorer event free survival in BCR-ABL1 positive cancers [158]. Synaptojanin, CTNND1, dynamin binding protein (DNMBP), Afadin, EPS15-like 1 (EPS15L1) were labeled as ABI1 proximal interactors (Fig. 2**AB**) [6]. ENL was not detected in ABI1 proximity proteomics. Several oncogenic MLL fusion partners appear to act in overlapping ABI1-mediated signalosomes involving actin cytoskeleton and cellular communication, supporting a role of ABI1 that affects leukemogenesis and identifying targetable signalosomes through association.

### ABI1 as a tumor suppressor

ABI1 is frequently reported to act as a tumor suppressor, as indicated by lower ABI1 expression observed in some types of cancer cells. In these cells, low ABI1 expression is associated with increased activity of signaling molecules driving malignant phenotypes including survival, proliferation, and invasiveness. Consistent with this, low ABI1 expression in some types of cancer is associated with negative patient outcomes. While ABI1 is also described as a tumor promoter, this section describes its reported role as a tumor suppressor in prostate, blood, brain, gastric, and colorectal cancers. Also included is a brief note on alternatively spliced forms of *ABI1* observed in cancer and their contributions to tumor suppression. Studies describing direct correlations of ABI1 expression level with cancer, which are cited in this section and the following section, “ABI1 as a tumor promoter”, are summarized in Table 2.

**Table 2:** Reported tumor suppressor and tumor promoting roles of ABI1 in various type of cancer

Cancer	ABI1 role	Reference
Brain	Tumor suppressor	Liu 2018
		Kumar 2015
Colorectal	Tumor suppressor	Li 2021
		Zhang 2021
		Baba 2012
Gastric	Tumor suppressor	Lin 2020
		Cui 2010
Myeloid	Tumor suppressor	Sun 2008
		Chorzalska 2014
		Chorzalska 2018
		Petersen 2023
Prostate	Tumor suppressor	Xiong 2012
		Nath 2019
		Macoska 2001
Breast	Tumor promoter	Wang 2007
		Wang 2011
		Sun 2009
		Regua 2022
Colorectal	Tumor promoter	Steinestel 2012
		Steinestel 2014
Liver	Tumor promoter	Wang 2017
		Xuan 2020
Lymphoid	Tumor promoter	Juskevicius 2016
Ovarian	Tumor promoter	Zhang 2015
		Chen 2010
		Yu 2019
		Fang 2017
Pancreatic	Tumor promoter	Tod 2017

In prostate tumors, loss of ABI1 is correlated with loss of heterozygosity (LOH) at markers of the long arm of chromosome 10. Low ABI1 expression is detected in the LNCaP human prostate cancer cell line, and ABI1 mutations are observed in prostate cancers. Dysregulated ABI1 in prostate tumors is linked to abnormal cell adhesion leading to prostatic intraepithelial neoplasia, characterized by increased AKT activation and decreased levels of E-cadherin, β-catenin, and WAVE2 [125]. Furthermore, disruption of ABI1 in a benign epithelial prostate cancer cell line, RWPE-1, results in increased invasion and loss of cell-cell adhesion markers. Additionally, a study of 505 patients with prostate cancer found that low ABI1 expression is associated with increased cancer recurrence, metastasis, and death, linked to activation of epithelial-mesenchymal transition (EMT) pathways and non-canonical WNT signaling. ABI1 can also inhibit EMT in prostate cancer by suppressing FYN-signal transducer and activator of transcription 3 (STAT3) activation by non-canonical WNT signaling through a high affinity interaction between the FYN SH2 domain and ABI1 pY421 [95].

FYN promotes WRC-mediated cell migration by phosphorylating and inactivating SKAP2 [124]. SKAP2 deficiency in murine colorectal cancer is associated with increased tumorigenesis linked to increased LPS-induced macrophage NF-κB activation [159]. While FYN was not detected in ABI1 proximity proteomics, FYN-binding protein 1 (FYB1) was reported as an ABI1 proximal interactor. FYB1 is a known interactor of SKAP2 [160], further supporting a role of ABI1 in this complex that communicates responses between cytoskeletal reorganization and cytoplasmic signaling. STAT3 was enriched in ABI1 proximity proteomics but was not significant.

WNT and NF-κB signaling through TNFR regulation also promote colorectal cancer progression in a mode coordinated by Disheveled segment polarity protein 2 (DVL2) [161]. Of note, DVL2 has multiple PRR and PXXP motifs that might facilitate ABI1 binding. E3 ubiquitin ligase Itchy homolog (ITCH) binds DVL2 and directs its degradation to inhibit canonical Wnt signaling, dependent on DVL2 PPXY and Disheveled, Egl-10, and Pleckstrin (DEP) domains [162]. ITCH is also required for c-Jun N-terminal kinase (JNK)-activated FLICE-like inhibitory protein (c-FLIP) degradation and TNF⍺-induced cell death [163]. Furthermore, DVL2 PSD95, Dlg1, and zo-1 (PDZ) domain is required to activate JNK, resulting in microtubule stability [164]. JNK is also activated by integrin signaling via p130Cas, FAK, and Crk [165], and crosstalk between FAK and WNT signaling is observed in cancer [166]. Together, these studies suggest a direct binding role of ABI1 in regulating cell fate through TNFR-JNK-WNT signaling. This is further supported by findings that ABI1 deficiency leads to altered RIPK1 phosphorylation and protection from cell death linked to TAK1-RIPK1 [6]. In this model, ABI1 may link actin and integrin signaling to RIPK1 and TNFR1-associated DEATH domain protein (TRADD), coordinating activation of ITCH toward JNK to promote c-FLIP degradation leading to caspase activation, or toward DVL2 degradation leading to non-canonical Wnt signaling. Furthermore, MAP kinase-activating death domain protein (MADD) is involved in growth factor receptor-bound protein 2 (GRB2) and SOS1 recruitment to activated TNFR, leading to GSK3β inhibition through RAS-RAF-mitogen-activated protein kinase kinase (MEK)-ERK signaling, decreased β-catenin nuclear translocation, and EMT gene transcription activation [167]. Recruitment of GRB2 and SOS1 to TNFR may couple this protein complex to PI3K-associated cytoskeleton regulation through ABI1. Several WNT/catenin pathway proteins were identified as ABI1 proximal interactors, including CTNND1, adenomatous polyposis coil (APC), and GSK3β (Fig. 2**AB**).

Altogether, these studies demonstrate how complex pathway crosstalk dynamically determines cellular signaling outcome. Integration of signaling and cellular response might be mediated through adaptor proteins such as ABI1. Towards this, ABI1 is observed to coordinate outcomes of integrin and cytoskeletal signaling, TNFR/NF-κB survival or death signaling, STAT signaling, WNT signaling, and JNK activation. Through disrupted communications among these mechanisms, loss of ABI1 may promote malignancy by EMT gene activation, actin cytoskeleton dysregulation, and inhibition of cell death signaling.

ABI1 also plays a role in regulation of reactive oxygen species production. Murine knockout of neutrophil cytosolic factor 1 (NCF1/p47phox), an adaptor protein involved in nicotinamide adenine dinucleotide phosphate (NADPH) oxidase activation, protects mice from colon cancer [168]. NCF1 is activated through binding ABL1 and ABI1 via its p47phox domain (Fig. 1A), producing reactive oxygen species (ROS) that increase ABL1 phosphorylation. Notably, murine ABI1-205 protein sequence does not show conservation with human ABI1 p47phox domain (Fig. 1B). Exogenous NCF1 overexpression in colon cancer cells increases oxidant production and nuclear ABL phosphorylation, leading to apoptosis [169]. NADPH oxidase localizes to ECM-degrading invadopodia in a human colorectal carcinoma cell line, and overexpression of p47phox family member NADPH oxidase organizer 1 (NOXO1) reduces ROS-positive invadopodia formation and ECM degradation [170]. These studies indicate that ABI1, along with other adaptors, might suppress ROS production and tumorigenesis by competitive binding with p47phox to inhibit its interaction with ABL1.

As mentioned, ABI1 is also downregulated in patients with myeloproliferative neoplasms (MPN), a class of blood cancers characterized by abnormal expansion of the myeloid blood compartment. CD34^+^ hematopoietic stem/progenitors and granulocytes from patients with a subtype of MPN – PMF – show decreased ABI1 expression, associated with increased activation of SFKs, STAT3, and NF-κB. Furthermore, murine bone marrow specific depletion of ABI1 causes MPN-like phenotype with similarities to PMF. ABI1-deficient murine bone marrow shows increased activity of AKT/ERK, SFK, STAT3, and NF-κB. Hematopoietic stem cells isolated from these mice have impaired self-renewal and fitness, consistent with MPN [141].

In neuroblastoma, siRNA against ABI1 results in more metastatic cells, and ABI1 overexpression reduces neuroblastoma cell migration. Aberrant miRNA regulation is frequently observed in neuroblastoma cells, and the miR-181 family was detected in 32 neuroblastoma patients. The 3’-untranslated region (UTR) of *ABI1* is targeted by miR-181a and miR-181b, resulting in decreased *ABI1* expression and increased neuroblastoma growth and metastasis [171]. Interestingly, miR-181a-mediated ABI1 deficiency is also observed in imatinib-resistant K562 cells, linking this mechanism of ABI1 regulation to CML [41]. CRK pY251, identified as a marker for aggressive glioblastoma correlated with high invasion and poor survival outcome, is negatively regulated by ABI1. ABI1 competes with CRK binding to ABL1, resulting in reduced ABL1 transactivation and decreased RAC1-mediated motility [172]. Glioblastoma migration affected by WAVE2, SKAP2, cortactin, and FYN was also discussed earlier in relation to ABI1 and ABL1, linking EGFR and SFK signaling directly to ARP2/3-mediated actin polymerization. Together, ABI1 can repress growth and metastasis of neuroblastoma cells through suppression of mechanisms that promote activation of downstream effectors including ABL1, and this can be regulated on the transcriptional level.

Finally, alternatively spliced forms of *ABI1* also have different effects on malignant transformation. Two prostate tumor cell lines carrying *ABI1* exon skipping mutations resulting in SH3 domain loss and consequential attenuation of ABL1 binding to ABI1 show loss of negative regulation of ABL1 by ABI1, resulting in increased ABL1 activation [173]. Another alternatively spliced form, ABI1-212, lacks the HHR, PXXP, and PRR but retains WAVE binding and SH3 domains [174]. ABI1-212 is a dominant-negative form of full-length ABI1 in colorectal cancer cells, competing with full length ABI1 to bind WAVE2 and phosphorylated full-length ABI1 to inhibit cell adhesion and migration. ABI1-212 is decreased in colorectal cancer cell lines and tissues, while full length ABI1 is elevated [175]. However, another study described ABI1-212 elevation in left-sided colorectal cancer, associated with increased lymph node metastasis and shorter overall survival. Overexpression of ABI1-212 results in increased cell adhesion, migration, and lung metastasis linked to altered actin dynamics through EPS8 interaction [174], consistent with loss of ABI1 tumor suppression capabilities. Furthermore, an *ABI1* exon skipping event in gastric cancers is associated with decreased overall and disease-free survival [176]. Decreased *ABI1* expression is also more frequently observed in gastric cancer, correlated with decreased survival time and five-year-survival rate [177]. *ABI1* isoforms are differentially downregulated in esophageal, gastro-intestinal, and colorectal cancers [178]. Disease outcome linked to ABI1 domain-affected signaling presents an informative window into how cross-regulation of essential cellular processes are affected on a protein interaction level. Together, these studies demonstrate how disruption of normal ABI1 functionality can promote malignancy through loss of negative regulation mechanisms mediated by ABI1 interaction.

### ABI1 as a tumor promoter

While reported as a tumor suppressor in the previous section, increased ABI1 expression is also associated with tumor metastasis, consistent with its role in regulating actin cytoskeleton organization. Upregulated ABI1 is observed in several different cancers, correlating with negative patient outcomes. For example, a study of 988 patients with invasive breast carcinoma found that ABI1 expression positively correlates with older age at diagnosis, earlier tumor recurrence, and lower survival linked to AKT activation [25], consistent with the role of ABI1 in PI3K signaling. Additionally, a study of mutation and copy number alteration in 20 paired relapsing and non-relapsing diffuse large B-cell lymphomas (DLBCL) found that a gain of *ABI1* is associated with relapsing DLBCL [179]. In different cancers, ABI1 promotes a mesenchymal-like phenotype and metastasis through RAC1 activation via the ABI1/SOS1/EPS8 complex. This section reviews molecular mechanisms by which overabundance of ABI1 promotes cancer cell motility and invasion in different types of cancer.

At the leading edge of motile cells, brain-specific angiogenesis inhibitor 1-associated protein 2 (BAIAP2/IRSp53) binds EPS8 to reinforce a RAC1 GTPase-activating complex comprising ABI1, EPS8, and SOS1. Inhibiting IRSp53/EPS8 complex formation suppresses invasiveness and motility of a fibrosarcoma cell line. IRSp53 is a known ABI1 interactor [180] and was also labeled as an ABI1 proximal interactor (Fig. 2**AB**) [6]. EPS8 is upregulated in most pancreatic ductal adenocarcinomas (PDAC). Downregulation of EPS8, SOS1, or RAC1 in PDAC cells suppresses cell movement but also increases integrin αVβ6-dependent TGFβ activation through Rho activation, which has both tumor suppressing and activating effects. Integrin αVβ6, which also promotes tumor invasion, is upregulated in adenocarcinomas and correlates with poor prognosis. The presence or absence of the EPS8/SOS1/ABI1 complex acts as a molecular switch in PDAC cells to balance RAC1 and RhoA activation to promote tumor migration or TGFβ activation, respectively [181].

ABI1 also promotes breast cancer progression and metastasis. ABI1 is more abundant in highly invasive breast cancer cell lines, and downregulation of ABI1 by RNA interference results in decreased lamellipodia formation, decreased adhesion, decreased proliferation, and decreased migration and invasion linked to defective PI3K/AKT and WRC signaling [24, 25]. Furthermore, ABI1 localizes to invadopodia in MDA-MB-231 cells, and ABI1 knockdown is associated with decreased invadopodia formation and reduced ECM degradation linked to decreased activation of the SRC-inhibitor of DNA binding 1 (ID1)-MMP9 pathway, which leads to lower expression of MMP9. Additionally, ABI1 knockdown in MDA-MB-231 cells is associated with slower murine tumor xenograft growth [133]. An analysis of 1,903 breast cancer patients found that ABI1 overexpression is associated with more aggressive cancer [182]. Another analysis of 988 patients with invasive breast carcinoma identified ABI1 as a prognostic marker associated with decreased overall and disease-free survival [25]. ABI1 is also identified as a prognostic marker for breast cancer metastasis and shows a gene dose-response in mice positively correlated with number of pulmonary metastases [182]. As breast cancer prognosis is highly dependent on metastatic potential, the role of ABI1 in promoting cell migration offers mechanistic insight to both better understanding pathobiology and providing therapeutic direction.

Further underlining apparently contrasting roles of ABI1 in tumor regulation, a study of 95 colorectal cancer patients found that ABI1 expression is positively correlated with metastasis and was overexpressed in inflammatory mucosa, sessile serrated polyps, adenomas, and tubular adenomas. In these cancers, ABI1 expression is also positively correlated with KRAS mutation and is proposed to be an early marker for KRAS mutagenesis in hyperplastic polyps [183]. KRAS is a prominent proto-oncogene, mutated in nearly 40% of cancer cases [184]. KRAS activation is upstream of signaling processes directly affected by ABI1 including PI3K/AKT and Rac/CDC42 [185, 186], and also affects MAPK cascade and NF-κB activation [187, 188]. Together, this highlights cross-regulation of inflammatory signaling pathways in cancer that might uphold therapeutic resistance, and how adaptor proteins such as ABI1 interlink these processes. ABI1 is also upregulated by TNFα treatment in colorectal cancer cell lines, and ABI1 overexpression is abolished by PI3K inhibitor treatment in KRAS-transfected cells [183]. This may also involve a role of ABI1 in coordinating actin polymerization activation via GRB2-SOS1-MADD binding, in which ABI1 overexpression leads to increased PI3K-RAC activation [27, 167]. Furthermore, in a study of 56 colorectal cancer samples, ABI1 positively correlates with an invasive phenotype and is upregulated at the invasive edge. In a KRAS-mutated CHD1 cell line, ABI1 localizes to sites of ECM dissolution, and *ABI1* knockdown by RNA interference suppresses matrix dissolution, invasion, and fibronectin attachment. This less invasive phenotype is also observed upon treatment with imatinib, which abolishes phosphorylation of ABI1 Y435 [189]. Together, these studies suggest an invasion-promoting role of ABI1 through inflammatory activation of PI3K and ABL1-mediated ABI1 Y435 phosphorylation.

ABI1 expression level may also have prognostic significance in ovarian cancer. A study of 46 patients with epithelial ovarian cancer found that ABI1 protein and mRNA expression are higher in cancerous tissue than in non-cancerous tissue. High ABI1 expression is correlated with shorter survival, increased cell invasiveness, more advanced-stage and higher-grade tumor, increased cancer antigen level, and suboptimal surgical debulking. ABI1 is posited to be an independent prognostic factor – as the tumor promoting gene – in epithelial ovarian cancer progression [190]. Lysophosphatidic acid (LPA) activates Ras, which activates RAC1 to induce lamellipodia formation in metastatic ovarian cancer cell lines but not in non-metastatic cell lines. Silencing expression of any members of the SOS1/EPS8/ABI1 tricomplex through shRNA blocks LPA-induced RAC activation [191]. Using short inhibitory peptides, Yu et al. [192] identified regions of ABI1 responsible for EPS8 and SOS1 binding in response to LPA. ABI1 binds EPS8 through its polyproline region and binds SOS1 through its SH3 domain. Blocking interaction between ABI1 and EPS8 in vivo suppresses LPA-induced invasion and metastasis of ovarian cancer cells [192]. EMT in ovarian cancer cells is dependent on RAC1 activation through the SOS1/EPS8/ABI1 complex, associated with increased MEK-ERK and SRC activation. Additionally, combined treatment with MEK1/2 and SRC inhibitors suppresses development of xenografts and prolonged survival of mice with ovarian cancer. Knockdown of SOS1, EPS8, or ABI1 decreases expression of vimentin and increases expression of E-cadherin, indicating loss of metastatic potential [193]. Interestingly, this is inconsistent with the earlier discussed role of ABI1 as a tumor suppressor in prostate cancer, in which decreased expression leads to decreased E-cadherin expression and inhibition of canonical WNT signaling [125]. In sum, ABI1 is essential to promote metastasis in ovarian cancer through SOS1/EPS8/ABI1 complex activation, linked to regulations of inflammatory signaling and cytoskeletal reorganization.

ABI1 upregulation in hepatocellular carcinoma (HCC) also positively correlates with tumor size, stage, number, and encapsulation. ABI1 expression is associated with shorter survival time and higher frequency of tumor recurrence. In vitro, ABI1 overexpression is associated with increased HCC cell proliferation, migration, and invasion. This is further supported by a xenograft mouse model that shows increased HCC growth and lung metastases with ABI1 overexpression. Consistently, ABI1 knockdown is associated with decreased HCC cell proliferation, migration, and invasion [194]. EPS8L3 also plays a role in HCC through modulation of EGFR dimerization/internalization and EGFR-ERK pathway activation. This is proposed to depend on formation of a protein complex comprising EPS8L3, SOS1, and ABI1. EPS8L3, which is overexpressed in HCC tissues, promotes cell proliferation through p21/p27 downregulation, and promotes migration and invasion through upregulation of MMP2 [195]. Similar to its metastasis-promoting role in ovarian cancer, ABI1 likely promotes metastasis in HCC as an essential component of the ABI1/EPS8L3/SOS1 complex.

Altogether, ABI1 overexpression in cancer is associated with a mesenchymal phenotype, characterized by increased migration and matrix dissolution, promoting invasion and metastasis of tumor cells. ABI1 overexpression is linked to negative cancer patient outcomes, including poorer prognosis and higher frequency of relapse. As an adaptor protein with divergent signaling properties, ABI1 plays an essential role in cellular homeostasis that, when dysregulated, can promote malignancy.

## Conclusions

ABI1 is an adaptor protein that coordinates several protein complexes associated with disease. While ABI1 is a prominent regulator of actin cytoskeleton and is essential for basic organismal processes such as development and smooth muscle contraction, the roles ABI1 plays in coordinating cytoplasmic protein interactions have become apparent as critical mediators of pathological protein signaling. ABI1 upholds signaling homeostasis between cellular environment and response. As such, ABI1 dysregulation is both directly and indirectly associated with disease, including pathogen infection and cancer. In addition to known roles of ABI1 in regulating actin cytoskeleton, EGFR, integrin, and vesicle transport signaling (Fig. 4**ABCD**), proximity proteomics and literature review support roles of ABI1 in centrosome regulation, TNFR signaling, and WNT signaling (Fig. 4**EFG**). Furthermore, aggregate interaction analyses of these identified ABI1-affected proteins indicate robust and extensive interconnectivity of biological processes (Fig. 4**HI**), supporting their functional or physical interactions with ABI1. Together, this highlights gaps in understanding complex intricacies of protein signal transduction, how cells maintain tight yet dynamic control over response, and how seemingly small perturbations in signaling may lead to broad disease outcomes. Interactome analysis of adaptor proteins such as ABI1 informs not only activities of the bait protein itself, but also of adjacent signaling processes that affect disease. This holistic philosophy of cellular signaling may hold the key to answering major biomedical challenges such as individual variability in treatment response, development of therapeutic resistance, and chronic inflammation. The combination of proximity proteomics and extensive literature analysis used in this report describes a strategy to detail the mechanistic complexity of cellular regulation, leading a more thorough future understanding of protein signaling in disease.

## Data Availability

ABI1 proximity proteomics data used throughout this manuscript are available and further described in Petersen *et al, Mol. Onc.,* 2023 [6]. An RShiny web app is also available as a user interface to explore and interpret this data (https://maxpetersen.shinyapps.io/turboabi_data_ui_v2/).

## Abbreviations

ABI1/E3B1/SSH3BP1: Abelson interactor 1
VASP: Vasodilator-stimulated phosphoprotein
WAVE: Wiskott-Aldrich syndrome protein family
N-WASP: Neural-Wiskott-Aldrich syndrome protein
ABL: Abelson kinase
CML: Chronic myeloid leukemia
EGFR: Epidermal growth factor receptor
EPS8: EGFR pathway substrate 8
SH3: Src homology 3
PRR: Proline-rich region
PXXP: Proline-X-X-proline
HHR: Homeodomain homologous region
T-SNARE: Target-soluble N-ethylmaleimide-sensitive factor attachment protein receptor
GTPase: Guanosine triphosphate hydrolase
Ras: Rat sarcoma virus
RAC1: Ras-related C3 botulinum toxin substrate 1
CDC42: Cell division control protein 42 homolog
ARP2/3: Actin-related protein 2/3
Dia: Diaphanous-related
WRC: WAVE regulatory complex
CYFIP1: Cytoplasmic fragile X messenger ribonucleoprotein 1 (FMR1)-interacting protein 1
BRK1: BRICK1 subunit of suppressor of cyclic adenosine monophosphate receptor (cAR) (SCAR)/WAVE actin nucleating complex
NCKAP1/NAP1: Non-catalytic region of tyrosine kinase adaptor protein (NCK) associated protein 1
PIP2: Phosphatidylinositol 4,5-bisphosphate
ENAH: Enabled homolog
EVL: Ena/VASP-like
DIAPH1/mDia: Protein diaphanous homolog 1
DAAM1: Disheveled-associated activator of morphogenesis 1
SOS1: Son of sevenless homolog 1
PI3K: Phosphoinositide 3-kinase
AKT: Protein kinase B
PIP3: Phosphatidylinositol (3,4,5)-triphosphate
GSK3β: Glycogen synthase kinase 3 beta
CBL: Casitas B-lineage lymphoma
ITSN1: Intersectin 1
IQGAP1: IQ motif containing GTPase activating protein 1
PH: Pleckstrin homology
SEC: Protein transport protein
ARF1: Adenosine diphosphate (ADP)-ribosylation factor 1
ARHGEF7/β-PIX: Rho guanine nucleotide exchange factor 7
GIT1/2 ARF: GTPase-activating protein 1/2
ARFGAP3: ADP ribosylation factor GTPase activating protein 3
SMAP2: Stromal membrane-associated protein 2
DOCK1: Dedicator of cytokinesis protein 1
BCAR1/p130Cas: Breast cancer resistance protein 1
BCR: Breakpoint cluster region
ITGA: Integrin subunit alpha
ITGB: Integrin subunit beta
PXN: Paxillin
GEF: Guanine nucleotide exchange factor
ARNO: Cytohesin 2
MEF: Mouse embryonic fibroblast
TOCA: Transducer of CDC42-dependent actin assembly
FNBP1L: Formin-binding protein 1like
FNBP1: Formin-binding protein 1
FMNL1: Formin-like protein 1
FAK/PTK2: Focal adhesion kinase
mTOR: Mammalian target of rapamycin
DTC: Distal tip cell
CED-1: Cell death abnormality protein 1
SCARF1: Scavenger receptor class F member 1
SLI-1: Suppressor of lineage defect 1
SK3: Small conductance calcium-activated potassium channel 3
SHANK: SH3 and multiple ankyrin repeat domains 3
CaMKIIa: Calcium/calmodulin-dependent protein kinase II-alpha
NMDA: N-methyl-D-aspartate
LPD: Lamellipodin
RAP1: Ras-related protein 1
RIAM: RAP1-GTP-interacting adaptor molecule
MRL: MIG-10/LPD/RIAM
UNC-53: Uncoordinated-53
NAV2: Neuron navigator 2
TARP: Translocated actin recruiting protein
HCMV: Human cytomegalovirus
ECM: Extracellular matrix
SH3KBP1: SH3-domain kinase-binding protein 1
PP2A: Protein phosphatase 2 A
AnkA: Ankyrin-rich protein
HCV: Hepatitis C virus
NS5A: Nonstructural protein 5 A
ERK: Extracellular signal-regulated kinase
EGR1: Early growth response factor 1
HASM: Human airway smooth muscle
BCAR3: Breast cancer anti-estrogen resistance protein 3
C3G: Rap guanine nucleotide exchange factor 1
CRKL: Crk-like protein
PIK3AP1: Phosphoinositide 3-kinase adaptor protein 1
SFK: SRC family kinases
SKAP2: Src kinase-associated phosphoprotein 2
IL-3: Interleukin-3
SDF1a: Stromal cell-derived factor 1 alpha
NOD/SCID: Nonobese diabetic/severe combined immunodeficiency
MT1-MMP: Membrane type 1 matrix metalloproteinase
MMP9: Matrix metalloproteinase 9
CDC2: Cell division cycle protein 2 homolog/cyclin-dependent kinase 1
BUB1: Budding uninhibited by benzimidazoles 1
PCM1: Pericentriolar material 1
HSBP1: Heat shock binding factor protein 1
WASHC3/CCDC53: Coiled-coil domain containing 53
WASH: Wiskott-Aldrich syndrome protein and SCAR homolog
PLK4: Polo-like kinase 4
CEP131: Centrosomal protein 131
OFD1: Oral-facial-digital syndrome 1
HAUS: Human Augmin-like complex subunit
NF-κB: Nuclear factor kappa-light-chain-enhancer of activated B cells
MAP3K7/TAK1: Mitogen activated protein kinase kinase kinase 7/transforming growth factor beta (TGFβ)-activated kinase
TNFα: Tumor necrosis factor alpha
TAB1: TAK1-binding protein 1
TAB2: TAK1-binding protein 2
IKK: Inhibitor of κB kinase
RIPK1: Receptor interacting serine/threonine-protein kinase 1
MLL1: Mixed-lineage leukemia 1
KMT2A: Lysine [K]-specific methyl transferase 2 A
AML: Acute myeloid leukemia
EEN: Extra eleven nineteen
EPS15: Epidermal growth factor receptor substrate 15
MLLT1/ENL: Mixed-lineage leukemia; translocated to, 1
CTNND1: Catenin delta-1
DNMBP: Dynamin binding protein
EPS15L1: EPS15-like 1
LOH: Loss of heterozygosity
EMT: Epithelial-mesenchymal transition
STAT3: Signal transducer and activator of transcription 3
FYB1: FYN-binding protein 1
DVL2: Disheveled segment polarity protein 2
ITCH: Itchy homolog
DEP: Disheveled, Egl-10, and Pleckstrin
JNK: c-Jun N-terminal kinase
c-FLIP: FLICE-like inhibitory protein
PDZ: PSD95, Dlg1, and zo-1
TRADD: TNFR1-associated DEATH domain protein
MADD: MAP kinase-activating death domain protein
GRB2: Growth factor receptor-bound protein 2
MEK: Mitogen-activated protein kinase kinase
APC: Adenomatous polyposis coil
NCF1/p47phox: Neutrophil cytosolic factor 1
NADPH: Nicotinamide adenine dinucleotide phosphate
ROS: Reactive oxygen species
NOXO1: NADPH oxidase organizer 1
MPN: Myeloproliferative neoplasm
PMF: Primary myelofibrosis
UTR: Untranslated region
DLBCL: Diffuse large B-cell lymphoma
BAIAP2/IRSp53: Brain-specific angiogenesis inhibitor 1-associated protein 2
PDAC: Pancreatic ductal adenocarcinoma
ID1: Inhibitor of DNA binding 1
LPA: Lysophosphatidic acid
HCC: Hepatocellular carcinoma
IS: Interaction score
GEO: Gene Expression Omnibus
PA: Protein abundance
PSM: Peptide spectrum match
FDR: False discovery rate
WAB: WAVE binding domain
COSMIC: Catalog of Somatic Mutations in Cancer
HPA: Human Protein Atlas
TCGA: The Cancer Genome Atlas
GENT2: Gene Expression database of Normal and Tumor tissues 2
CNV: Copy number variation
